# Epidermal activation of Hedgehog signaling establishes an immunosuppressive microenvironment in basal cell carcinoma by modulating skin immunity

**DOI:** 10.1002/1878-0261.12758

**Published:** 2020-07-21

**Authors:** Sandra Grund‐Gröschke, Daniela Ortner, Antal B. Szenes‐Nagy, Nadja Zaborsky, Richard Weiss, Daniel Neureiter, Martin Wipplinger, Angela Risch, Peter Hammerl, Richard Greil, Maria Sibilia, Iris K. Gratz, Patrizia Stoitzner, Fritz Aberger

**Affiliations:** ^1^ Department of Biosciences Cancer Cluster Salzburg Paris‐Lodron University Salzburg Austria; ^2^ Department of Dermatology Venereology & Allergology Medical University Innsbruck Austria; ^3^ IIIrd Medical Department Salzburg Cancer Research Institute ‐ Laboratory for Immunological and Molecular Cancer Research (SCRI‐LIMCR) Paracelsus Medical University Salzburg Cancer Cluster Salzburg Austria; ^4^ Institute of Pathology Paracelsus Medical University Salzburg Cancer Cluster Salzburg Austria; ^5^ Institute of Cancer Research Comprehensive Cancer Center Medical University of Vienna Austria

**Keywords:** basal cell carcinoma, cancer immunotherapy, hedgehog/gli signaling, immune checkpoint molecules, neutrophils, regulatory T cells

## Abstract

Genetic activation of hedgehog/glioma‐associated oncogene homolog (HH/GLI) signaling causes basal cell carcinoma (BCC), a very frequent nonmelanoma skin cancer. Small molecule targeting of the essential HH effector Smoothened (SMO) has proven an effective therapy of BCC, though the frequent development of drug resistance poses major challenges to anti‐HH treatments. In light of recent breakthroughs in cancer immunotherapy, we analyzed the possible immunosuppressive mechanisms in HH/GLI‐induced BCC in detail. Using a genetic mouse model of BCC, we identified profound differences in the infiltration of BCC lesions with cells of the adaptive and innate immune system. Epidermal activation of Hh/Gli signaling led to an accumulation of immunosuppressive regulatory T cells, and to an increased expression of immune checkpoint molecules including programmed death (PD)‐1/PD‐ligand 1. Anti‐PD‐1 monotherapy, however, did not reduce tumor growth, presumably due to the lack of immunogenic mutations in common BCC mouse models, as shown by whole‐exome sequencing. BCC lesions also displayed a marked infiltration with neutrophils, the depletion of which unexpectedly promoted BCC growth. The study provides a comprehensive survey of and novel insights into the immune status of murine BCC and serves as a basis for the design of efficacious rational combination treatments. This study also underlines the need for predictive immunogenic mouse models of BCC to evaluate the efficacy of immunotherapeutic strategies *in vivo*.

## Graphical abstract

Activation of hedgehog/glioma‐associated oncogene homolog (HH/GLI) signaling induces basal cell carcinoma (BCC) and establishes an immunosuppressive tumor microenvironment. HH/GLI signaling inhibits the activity of antitumoral T cells *via* programmed death ligand 1/programmed death‐1 immune checkpoint signaling and the recruitment of immunosuppressive regulatory T cells. BCCs also show strong infiltration with antitumoral neutrophils. The data support the evaluation of combination treatments with HH inhibitors and immune checkpoint blockers.

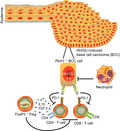

**Abbreviations**
BCC: basal cell carcinoma
DETC: dendritic epidermal T cell
GLI: glioma‐associated oncogene homolog
HH: hedgehog
MDSC: myeloid‐derived suppressor cell
NK: natural killer
PD: programmed death
PD‐L: programmed death ligand
PTCH1: Patched‐1
SMO: Smoothened
TAM: tamoxifen
TGF: transforming growth factor
Treg: regulatory T cells

## 1 Introduction

Basal cell carcinoma (BCC) of the skin represents a very common human cancer entity with around 3–4 million new cases diagnosed per year in the United States alone [1]. The etiology of BCC is based on the persistent ligand‐independent activation of hedgehog/glioma‐associated oncogene homolog (HH/GLI) signaling, caused in the vast majority of cases by inactivating mutations in the HH receptor and pathway repressor Patched‐1 (PTCH1) or in fewer cases by activating mutations in the essential HH effector Smoothened (SMO). Under normal physiological conditions, HH/GLI signaling is actively repressed by PTCH1 in the absence of HH ligand. Pathway activation occurs upon binding of secreted HH protein to its receptor PTCH1, a twelve‐transmembrane domain protein. Ligand binding results in the endocytic uptake of the PTCH1‐HH complex, thereby allowing SMO—a G protein‐coupled receptor‐like transmembrane protein—to enter the primary cilium. Activation of SMO within the primary cilium triggers the formation of activator forms of GLI zinc‐finger transcription factors. In response to their activation, GLI proteins translocate to the nucleus to induce HH target gene expression. In BCC, irreversible activation of HH signaling culminates in high levels of oncogenic GLI transcription factors, which initiate and promote tumor growth by continuous hyperactivation of HH target genes involved in proliferation, survival, angiogenesis, stemness and (de‐) differentiation [2, 3, 4, 5, 6, 7, 8, 9, 10, 11, 12]. Although most BCC lesions are routinely removed by surgical excision, unresectable locally advanced and metastatic BCC require drug therapy. Small molecule inhibitors targeting SMO such as vismodegib and sonidegib have shown therapeutic efficacy in locally advanced and metastatic BCC, with overall response rates of 40–60 percent and complete responses in about 20 percent of patients [13, 14, 15, 16, 17]. However, despite the striking therapeutic efficacy of SMO inhibitors, their successful clinical use is limited and challenged by frequent *a priori* and acquired drug resistance, lack of durable responses, severe adverse effects and relapse of patients upon drug withdrawal. These limitations call for novel therapeutic regimens improving the response rates and durability of the therapeutic effect of HH inhibitors [1, 18, 19, 20, 21, 22].

The recent breakthroughs in cancer immunotherapy that are based on our present understanding of how cancer cells evade the surveillance machinery of the adaptive and innate immune system have guided and paved the way to more efficacious, durable and even curative cancer therapies [23]. For instance, therapeutic antibodies targeting immune checkpoints such as programmed death‐1/programmed death‐ligand 1 (PD‐1/PD‐L1) signaling have been shown to re‐instate the antitumoral immune response resulting in yet unprecedented therapeutic efficacy even in metastatic diseases [24, 25, 26]. Intriguingly, two reports have already demonstrated a therapeutic benefit of anti‐PD‐1 treatment for BCC patients and a combination of vismodegib and pembrolizumab is currently evaluated in a clinical trial (trial ID: NCT02690948) [27, 28]. Together, these data raise the hope that rational combination treatments targeting oncogenic HH/GLI and immunosuppressive mechanisms will synergistically improve the efficacy and durability of the therapeutic response of BCC patients with advanced or metastatic disease. A detailed understanding of the immune microenvironment of BCC as well as of the molecular players involved in establishing immune evasion is, therefore, of critical importance for the advancement of combination immunotherapy for unresectable BCC.

In this study, we strived to systematically investigate the molecular and cellular status of the immune microenvironment of HH/GLI‐induced BCC, using a mouse model mimicking the genetic etiology of human BCC. We demonstrate that epidermal activation of HH/GLI signaling entails a variety of immunomodulatory mechanisms known to suppress immune recognition and subsequent eradication of neoplastic cells, thereby providing a basis for future combination treatments including immunotherapeutic drugs. In addition, we performed genomic sequencing of murine BCC‐like lesions, which unlike in human BCC revealed no significant mutational burden, underlining the high need for predictive immunogenic murine BCC models to evaluate *in vivo* the efficacy of novel combinatorial immunotherapeutic treatments.

## 2 Material and methods

### 2.1 Mice

**Fig. 1:**
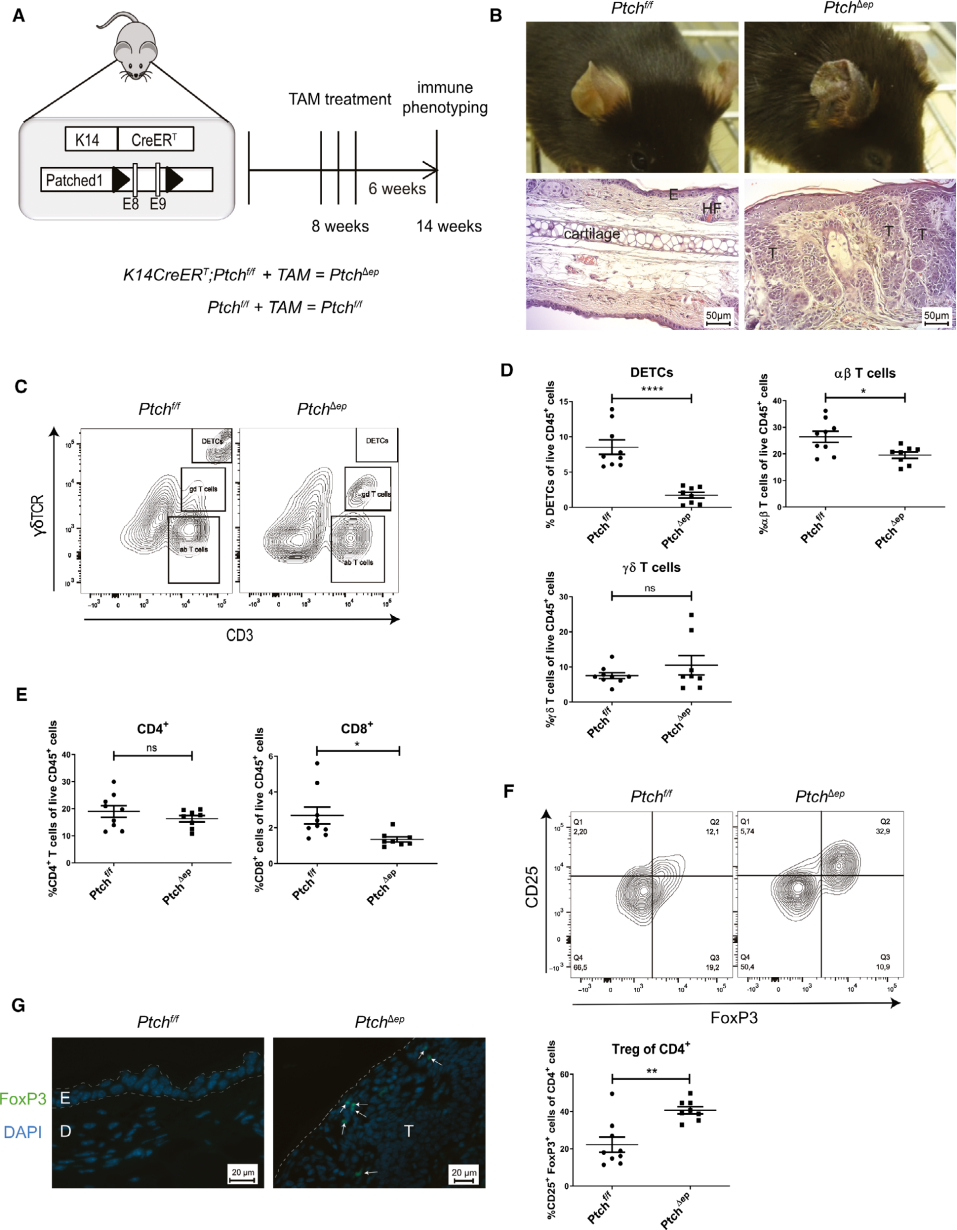
Oncogenic HH signaling leads to altered T cell populations in BCC‐like skin. (A) Schematic illustration of the *K14CreER^T^;Ptch^f/f^* (*Ptch^Δep^*) mouse model and TAM treatment schedule. (B) Representative figures of the phenotype (upper panel) and H&E stainings (lower panel) of *Ptch^f/f^* and *Ptch^Δep^* mouse ears (scale bar 50 µm). (C) Representative flow cytometry plots for T cell separation in mouse skin, using antibodies against γδTCR and CD3. (D–F) Flow cytometry analysis of (D) T cell populations, (E) CD4^+^ and CD8^+^ T cells and (F) CD25^+^ FoxP3^+^ Treg cells in *Ptch^Δep^* (*n* = 8) and *Ptch^f/f^* (*n* = 9) mouse skin. (F) Representative flow cytometry plot of CD25^+^ FoxP3^+^ Treg of CD4^+^ T cells in mouse skin (upper panel) and quantitative result of the flow cytometry analysis (lower panel). Cells were pregated for CD4. (G) Immunofluorescence analysis of mouse ear (*n* = 3) for FoxP3 (green) and DAPI (blue; scale bar 20 µm). The arrows indicate the FoxP3^+^ cells. D, dermis; E, epidermis, HF, hair follicle; T, tumor mass. Statistical analysis for all experiments was performed with Student's *t* test. *P < 0.05, **P < 0.01 and ****P < 0.0001.

*K14CreER^T^* (#005107) and *Ptch^f/f^* (#012457) mice were obtained from the Jackson Laboratory (Bar Harbor, ME, USA) and genotyped according to the supplier's instructions. For tumor induction, 8‐week‐old mice and Cre negative *Ptch^f/f^* animals were orally administered three times with 1 mg tamoxifen (TAM; Sigma, St Louis, MO, USA) dissolved in 10% ethanol/corn oil. Mice were analyzed 6 weeks after treatment, when they showed prominent pigmented lesions on the ears (also see Fig. 1A,B). All studies were performed on mice of both genders.

*R26SmoM2:YFP* [29] and *K5creER^T^* mice [30] were bred to obtain *K5creER^T^*;R26*SmoM2:YFP* (*SmoM2*) mice. Activation of *SmoM2* expression was accomplished by i.p. injection of 0.5 mg TAM citrate per day starting at postnatal day 14 for five consecutive days; Cre negative littermates received the same treatment. Mice started to develop macroscopically visible tumors 5 weeks after TAM administration.

For PD‐1 blocking, TAM‐treated *K14CreER^T^;Ptch^f/f^* mice were intraperitoneally injected every third day for 5 weeks with 250 µg of anti‐PD‐1 blocking antibodies (clone RMP1‐14, BioXCell, West Lebanon, NH, USA), according to previously published papers [31, 32, 33, 34]. A schematic overview is provided in Fig. 3A.

Neutrophils were depleted by intraperitoneal injection of anti‐Ly6G antibodies (clone 1A8; BioXCell). After TAM treatment, *K14CreER^T^;Ptch^f/f^* mice received an initial dose of 400 µg anti‐Ly6G followed by thrice weekly injections of 100 µg for 4 weeks, according to Coffelt *et al*. [35]. Successful depletion was verified weekly *via* flow cytometry staining of mouse blood. All studies were performed on mice of both genders.

Animal studies were performed in accordance with the institutional authorities and with the Federal Ministry of Science, Research and Economy (BMWFW‐66.012/0016‐WF/V/3b/2015 and BMWFW‐66.011/0030‐II/3b/2014).

### 2.2 Flow cytometry

For flow cytometry analysis of *K14CreER^T^;Ptch^f/f^* and control mice, the skin was digested for 45 min at 37 °C with 2 mg·mL^−1^ collagenase XI, 0.5 mg·mL^−1^ hyaluronidase und 0.1 mg·mL^−1^ DNase in Dulbecco's Modified Eagle Medium (all from Sigma). Skin cell suspensions were filtered through 70‐µm cell strainer (Corning, Corning, NY, USA). Cells were stained with directly conjugated antibodies for 30 min at 4 °C in the dark. Separation of dead cells was accomplished with fixable viability dye eFluor 780, eFluor 520 or eFluor 506 (dilution 1 : 1000; Thermo Fisher Scientific, Waltham, MA, USA). To separate immune cells from other skin cells, CD45 as pan‐leukocyte marker was included in all skin panels. Cells were fixed by using the Foxp3/transcription factor staining buffer set (Thermo Fisher Scientific) followed by intracellular staining. All experiments were performed on the BD FACS Canto II (BD Biosciences, Franklin Lakes, NJ, USA). For data analysis, the BD FACSDiva™ and the flowjo® software (BD Biosciences) were used.

For flow cytometry analysis of *SmoM2* mice, the ear skin was cut into small pieces and digested with 0.15 mg·mL^−1^ Liberase™ and 0.12 mg·mL^−1^ DNAse I (both Roche Diagnostics, Indianapolis, IN, USA) for 45 min at 37 °C and pressed through 100‐μm cell strainers (BD Biosciences). Nonspecific FcR‐mediated antibody staining was blocked with anti‐CD16/32 (2.4G2, in‐house from hybridoma supernatant or from BD Biosciences) for 15 min at 4 °C. Cells were stained with directly conjugated antibodies for 15 min at 4 °C in the dark. Dead cells were excluded with 7‐AAD (BD Biosciences) or fixable viability dye eFluor® 780 (Thermo Fisher Scientific). To separate immune cells from other skin cells, CD45 as pan‐leukocyte marker was included in all skin panels. Cells were fixed by using the Foxp3/transcription factor staining buffer set (Thermo Fisher Scientific) or the Cytofix/Cytoperm™ kit (BD Biosciences) followed by intracellular staining. All experiments were performed on the BD FACS Canto II (BD Biosciences). For data analysis, the flowjo® software (BD Biosciences) was used.

Antibodies used for flow cytometry are listed in Table S1.

### 2.3 Quantitative PCR

Total RNA isolation and qPCR analysis of mRNA expression was carried out as describes previously [36]. The quantity and quality of isolated RNAs were assessed by Agilent Bioanalyzer 2200 system (Agilent Technologies, Santa Clara, CA, USA). qPCR was done on Rotor‐Gene Q (Qiagen, Hilden, Germany) by using the GoTaq (Promega, Madsion, WI, USA) or the Luna (NEB, Ipswich, MA, USA) qPCR master mix. Details about primers used in the study are listed in Table S2.

Total RNA from *SmoM2* mouse skin was isolated with TRIZOL (Thermo Fisher Scientific). Genomic DNA was removed using the RapidOut DNA removal kit (Thermo Fisher Scientific). Random primed cDNA was prepared with the Superscript II RNase H‐reverse transcriptase (Thermo Fisher Scientific). mRNA expression was examined with quantitative PCR analysis by using the Brilliant III Ultra‐fast qPCR Master Mix (Agilent Technologies). Information about the primers used is listed in Table S3.

### 2.4 Luminex cytokine profiling

Mouse dorsal skin was homogenized with the Ultra‐Turrax® (IKA, Staufen, Germany) in PBS supplemented with protease inhibitor (Sigma) and filtered through Corning® Costar® Spin‐X® Plastic Centrifuge Tubes (Sigma). For cytokine profiling, the ProcartaPlex™ multiplex system (Thermo Fisher Scientific) was used. The array analyses were carried out according to the supplier's instructions. Measurement was performed on the Magpix instrument (Luminex corp, Austin, TX, USA), and the analysis was done with the procartaplex analyst 1.0 software (Thermo Fisher Scientific).

### 2.5 Histological analysis and immunofluorescence

Mouse ears were fixed overnight in 4% paraformaldehyde before paraffin embedding. 4‐µm sections from paraffin‐embedded tissue were prepared for hematoxylin and eosin and immunofluorescence staining. Before staining, samples were deparaffinized and sections were blocked for 1 h in 1% BSA. For immunofluorescence staining, slides were incubated overnight with antibodies against CD8, FoxP3, PD‐1 and Ly6G. For intracellular staining of FoxP3, Triton X‐100 (Sigma) was added to a final concentration of 0.1%. Alexa 488‐ or Alexa 555‐conjugated secondary antibodies were used for detection. Slides were mounted with Fluoroshield Mounting Medium with DAPI (Abcam, Cambridge, UK). Antibodies used for staining and detection are listed in Table S4. All pictures were taken on a Zeiss Axio Observer Z1 microscope (Carl Zeiss, Oberkochen, Deutschland) using zen 2.6 software (Carl Zeiss).

For quantification of the tumor area after neutrophil depletion, representative pictures from ears of three control and three tumor mice were quantified with the ImageJ software by three independent researchers in a blinded manner.

Immunohistochemistry of human BCC was done on FFPE tissue of eight different skin specimens with diagnosis of BCC [six female and two male patients, median age of 78.5 years and major localization of face (*n* = 5), trunk and extremities (*n* = 3)]. In brief, 4‐µm sections were mounted on glass slides, deparaffinized with graded alcohols, and stained using the primary antibodies listed in the Table S4, with pretreatment using pH 6.1 (FOXP3) or pH9 (all other antibodies) antigen retrieval buffer (Agilent Dako, Santa Clara, CA, USA). The immunohistochemical stainings were performed on a Benchmark Ultra platform with the OptiView (PD‐1 and PD‐L1) or Ultraview (CD4, CD8, CD15 and FOXP3) DAB IHC detection kit (Ventana, Tucson, AZ, USA). For double IHC stainings, the first (PD‐L1 or PD‐1) and second (PD‐L1: CD8 or PD‐1: CD4, CD8 and FOXP3) antibodies were sequentially detected with the DAB (brown color) and Fast‐Red (red) chromogen detection kit (Ventana).

### 2.6 Mouse whole‐exome sequencing

Mouse genomic DNA was isolated from ear tissue using the DNeasy Blood and Tissue kit (Qiagen). Isolated genomic DNA was sheared with the Covaris M220 (Covaris, Woburn, MA, USA). DNA was subjected to whole‐exome library generation [SureSelect^XT^ Mouse All Exon Kit; 49.6 megabases (Agilent)], which were sequenced 100 bp paired‐end on the Illumina platform NextSeq 550 using the NextSeq 500/550 v2.5 kit (Illumina, San Diego, CA, USA). Sequencing reads were mapped to mouse reference genome (UCSC mm10) using Burrows‐Wheeler Aligner with default settings (BWA‐MEM v0.7.12‐R1039) [37]. Duplicate removal was performed with default parameters using PicardTools (v2.10.3, http://broadinstitute.github.io/picard/). Alignments were preprocessed including local realignment around indels, and base quality recalibration was performed using Genome Analysis Tool Kit (GATKv3.7; all with default parameters) [38]. For somatic mutation calling (SNVs, Indels), each tumor sample (*K14CreER^T^;Ptch^f/f^*) was compared with three *Ptch^f/f^* mice. Subsequently, all unique SNVs and indels were called. Mpileup file generation was done by samtools (v1.5; parameter –B –q 1) [39], varscan2 (v2.4.3) was used for somatic variant calling (–min‐coverage‐normal 5 –min‐coverage‐tumor 5 –min‐var‐freq 0.05 ‐somatic‐*P*‐value 0.05 –strand‐filter 1), and filtering of high confidence calls was performed according to Basic Protocol 2 published by Koboldt *et al*. [40]. Variants were annotated using annovar (version 2017Jul16) [41]. All programs were executed following the authors' recommendations. Nonsynonymous exonic SNVs and Indels were considered. All remaining mutations were individually checked for accuracy using Integrative Genomics Viewer (igv) version 2.4.2. [42, 43]. For Copy Number Variations (CNVs) detection, depth of coverage was calculated for each exome target region using Varscan copynumber and Varscan copycaller. Coverage data were then analyzed using the r (version 3.6.1) package dnacopy (version 1.58.0) using the circular binary segmentation algorithm (CBS) to segment DNA copy number data and identify genomic regions with abnormal copy number [44].

### 2.7 Alternative activation of mouse bone marrow‐derived macrophages

Mouse bone marrow cells were isolated from the femoral bones and after red blood cell lysis grown in L929 fibroblast‐conditioned medium (L929‐sup, made in house) for 10 days at 7% CO_2._ Afterward, bone marrow macrophages were alternatively activated with 50 ng·mL^−1^ recombinant mouse IL4 (Immunotools, Friesoythe, Germany) for 24 h.

### 2.8 Statistical analysis

Significant differences between two groups were determined using an unpaired two‐tailed Student's *t* test. Values are given as means (± SEM) and were analyzed by graphpad prism® 8 (GraphPad, San Diego, CA, USA). For human BCC stainings, the statistical analysis was performed with the IBM Corperations (Armonk, NY, USA) ssps Statistics software. Here, the Levene test was used prior the *t* test. *P* values of < 0.05 were considered significant, and *P* values have been labeled and designated as follows: *P < 0.05, **P < 0.01, ***P < 0.001 and ****P < 0.0001.

## 3 Results

### 3.1 Oncogenic HH/GLI signaling alters T cell populations in mouse BCC‐like skin

The patients' response to cancer immunotherapy correlates with high mutational burden of the cancer tissue, presumably due to increased immunogenicity as a consequence of neoantigen expression [45, 46]. Intriguingly, genomic sequencing of human BCC revealed that HH/GLI‐driven skin cancers display an exceptionally high mutational burden with an average of 65 mutations per megabase [47], suggesting that BCC is likely to represent an immunogenic cancer entity. We, therefore, hypothesize that oncogenic HH/GLI signaling drives BCC growth by suppressing the antitumoral immune response and possibly, also by recruiting inflammatory cells with tumor‐promoting function. Although single case and prove‐of‐concept studies have suggested that immunotherapeutic approaches can be successful in BCC [27, 28], very little is known about how oncogenic HH/GLI signaling regulates tumor immunity in this cancer entity. To investigate the immunological modulation of BCC‐like tumors in mouse skin in response to ligand‐independent Hh/Gli activation, we treated 8‐week‐old *K14CreER^T^;Ptch^f/f^* (*Ptch^Δep^*) mice with TAM to induce irreversible Hh/Gli activation and BCC‐like tumor formation, respectively. TAM‐treated, Cre negative animals (*Ptch^f/f^*, controls) were used as controls (Fig. 1A). In agreement with previous reports [48, 49], mice with epidermal‐specific deletion of Ptch1 displayed a hyperproliferative BCC‐like phenotype that was most prominently observable on the ears (Fig. 1B).

To analyze alterations of adaptive immune cells in tumor lesions of *Ptch^Δep^* mice, we performed flow cytometry analysis of skin T cell populations. Viable CD45^+^ cells were gated for CD3 versus γδTCR, resulting in three distinct T cell populations with CD3^+^ γδTCR^‐^ cells representing αβ‐T cells, CD3^+^ γδTCR^+^ cells representing γδ‐T cells and cells that showed very high expression of both markers known as dendritic epidermal T cells (DETCs; Fig. 1C). Interestingly, αβ‐T cells and DETCs were decreased in *Ptch^Δep^* mice in comparison with TAM‐treated control mice, whereas the γδ‐T cells remained unchanged (Fig. 1D). We validated these results by analyzing *K5creER^T^*;*SmoM2* (*SmoM2*) BCC mice [36] expressing constitutively active oncogenic SMO in the epidermis after TAM injection. As shown in Fig. S1A, CD3^+^ and CD3^high^ (DETC) T cells were also reduced in lesional skin of *SmoM2* mice compared to controls (Fig. S1A).

Having shown that αβ‐T cells were decreased in skin tumors, we next addressed whether this applies to CD8^+^ or CD4^+^ T cell subsets. Gating for both markers revealed that cytotoxic CD8^+^ T cells were slightly lowered in the skin of tumor‐bearing mice, whereas the CD4^+^ T cell population stayed unaffected (Fig. 1E). Of note, this CD8 decrease could be confirmed in *SmoM2* BCC mice. However, in contrast to *Ptch^Δep^* mice CD4^+^ T cells were increased in *SmoM2* mice (Fig. S1B). Whether the differences between the two murine BCC models are a due to overexpression of the mutant SmoM2 oncogene, which itself might be immunogenic or a result of SMO‐independent roles of Ptch1 as dependence receptor [50] and binding partner cell cycle regulators such as Cyclin B [51] remains to be addressed. However, since nine out of 10 patients present inactivating mutations in PTCH1 [1], we focused on a detailed and comprehensive analysis of the Patched1‐deletion model of BCC.

Regulatory T cells (Treg) cells play a pivotal role in cancer immune evasion by suppressing the antitumoral immune response through different mechanisms such as the production of immunosuppressive cytokines [52, 53]. Although the percentages of CD4^+^ lymphocytes were not changed in *Ptch^Δep^* mice, we asked whether CD4^+^ FoxP3^+^ CD25^+^ Treg cells were altered. Notably, and as shown in Fig. 1F, FoxP3^+^ Tregs were strongly increased in mouse skin tumors compared to nonlesional control skin. We analyzed by qPCR *FoxP3* expression in SmoM2 mice and found elevated *FoxP3* transcript levels compared to control mice, suggesting that BCC lesions from SmoM2‐expressing mice comprise elevated Treg cell numbers similar to Ptch1‐deficient tumors (Fig. S1C). To determine the localization of Treg cells, we performed immunofluorescence staining of *Ptch^Δep^* mouse ears. We found Treg cells to be localized in intra‐ and peri‐tumoral regions, suggesting a possible role of Tregs in immunosuppression of the BCC microenvironment (Fig. 1G). We confirm Treg infiltration in the stromal compartment of human BCC and show that FoxP3^+^ cells do only rarely if at all co‐express with the immune checkpoint PD‐1 (Fig. S2A).

### 3.2 Increased immune checkpoint expression in mouse BCC‐like skin

**Fig. 2:**
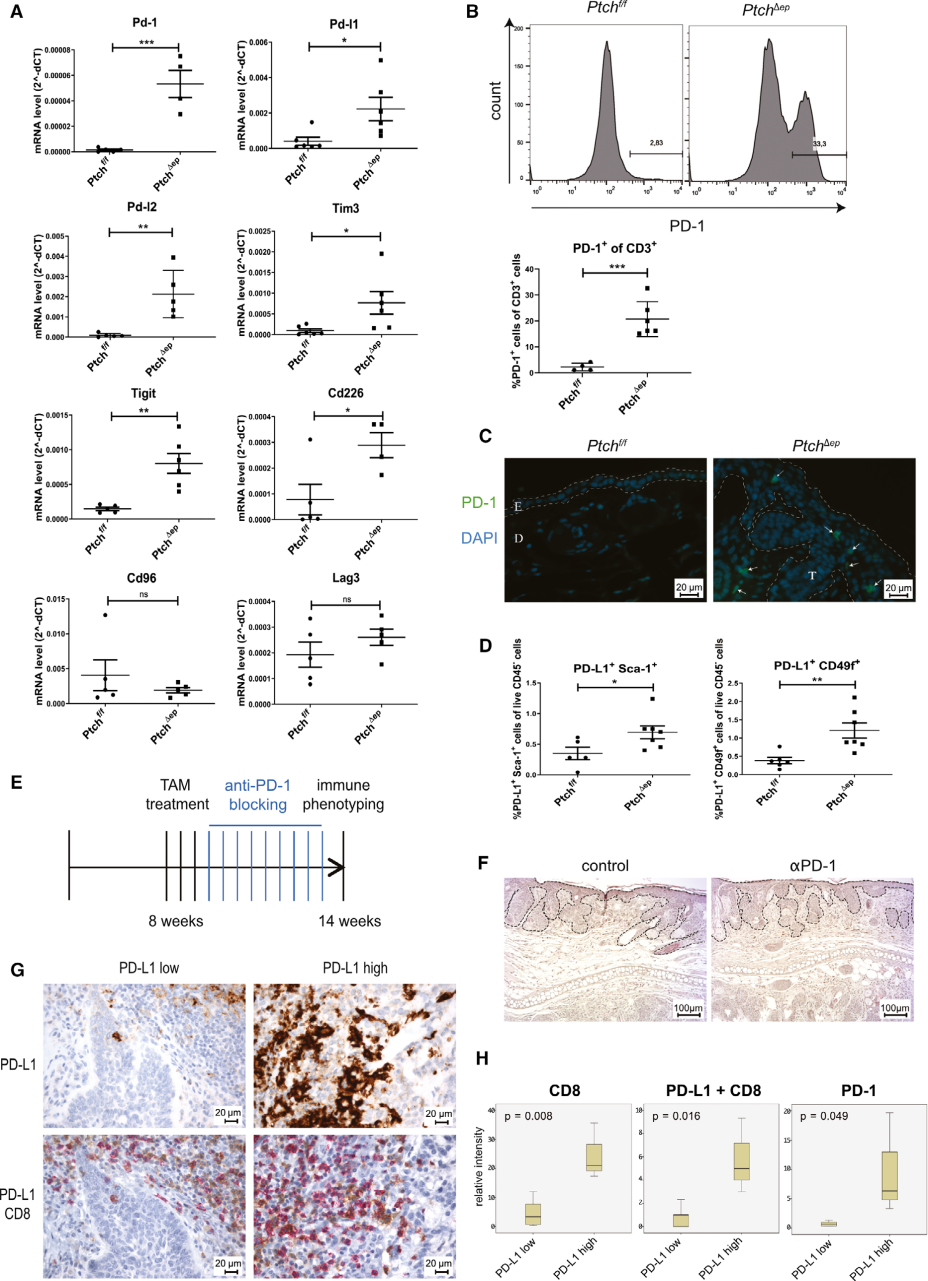
Increased immune checkpoint expression in BCC tumors. (A) qPCR analysis of mouse skin (*n* for *Pd‐1* and *Cd226: Ptch^f/f^* = 5 and *Ptch^Δep^* = 4, for *Pd‐l1* and *Tim3: Ptch^f/f^* = 6 and *Ptch^Δep^* = 6, for *Pd‐l2* and *CD96: Ptch^f/f^* = 5 and *Ptch^Δep^* = 5, and for *Tigit: Ptch^f/f^* = 5 and *Ptch^Δep^* = 6). (B) Representative flow cytometry plot showing PD‐1 expression on CD3^+^ T cells in mouse skin (upper panel) and flow cytometry analysis of PD‐1 expression on CD3^+^ T cells (lower panel; *Ptch^f/f^* *n* = 4 and Ptch*^Δep^* *n* = 6). Cells were pregated for CD3. (C) Immunofluorescence analysis of murine ear skin stained for PD‐1 (green) and DAPI (blue). The arrows highlight PD‐1^+^ cells (scale bar 20 µm). (D) Flow cytometry analysis of PD‐L1 expression on CD45^−^ Sca‐1^+^ (*Ptch^f/f^* *n* = 5 and Ptch*^Δep^* *n* = 7) and CD49f^+^ (*Ptch^f/f^* *n* = 6 and Ptch*^Δep^* *n* = 7) keratinocytes in mouse skin. (E) Treatment schedule of PD‐1 blocking in previously TAM‐treated *K14CreER^T^;Ptch^f/f^* BCC mice. (F) Representative images of H&E staining of ear skin of *Ptch^Δep^* mice treated with anti‐PD‐1 blocking antibody or untreated (*n* = 4 per group). The dashed lines mark the tumor area (scale bar 100 µm). (G) Representative immunohistochemical stainings of human BCC skin sections for PD‐L1 (brown) alone and together with CD8 (red) nuclei are stained in blue. The samples were divided by PD‐L1 low (*n* = 5) and high (*n* = 3) expression (scale bar 20 µm). (H) Quantification and statistical analysis of the CD8 and CD8 plus PD‐L1 and PD‐1 expression from PD‐L1 low (*n* = 5) and high (*n* = 3) BCC. D, dermis; E, epidermis; T, tumor mass. Statistical analysis was performed with Student's *t* test except for (H), which was analyzed with the Levene test to prior *t* test. For (H), the whiskers show the minimum and maximum of data points. *P < 0.05, **P < 0.01 and ***P < 0.001.

Having shown increased levels of immunosuppressive Tregs in mouse BCC‐like skin, we next addressed the question of whether additional immunoregulatory mechanisms may support tumor immune escape. To this end, we performed a systematic screen for known immune checkpoints [54] *via* quantitative PCR of mouse BCC‐like and control skin. Interestingly, we found *Pd‐1* and its ligands *Pd‐l1* and *Pd‐l2* as well as *Tigit*, *Tim3,* and *Cd226* to be significantly upregulated in skin tumors, whereas *Lag3* and *Cd96* were not significantly changed compared to control skin (Fig. 2A). In addition, we confirmed upregulation of *Pd‐l1* mRNA levels in tumor lesions of *SmoM2* mice (Fig. S1D).

Given the clinical relevance of the PD‐1/PD‐L1 axis in cancer immunotherapy [54, 55, 56, 57, 58], we analyzed PD‐1 expression on skin T cells of *Ptch^Δep^* and control mice. As shown in Fig. 2B, on average 20% of all T cells expressed PD‐1 in tumor lesions of *Ptch^Δep^* mice, whereas these cells were virtually absent in control skin (Fig. 2B). Flow cytometry analysis revealed that both CD4^+^ and CD8^+^ T cells express PD‐1, although the data did not reach statistical significance due to low mouse numbers in this analysis (Fig. S2B). To determine the *in situ* localization of PD‐1‐positive cells in the skin, we performed immunofluorescence staining of mouse ears. We found most PD‐1^+^ cells to be located adjacent or within the epithelial tumor compartment (Fig. 2C).

We next performed double stainings of human BCC samples for PD‐1 and either CD4 or CD8. We show that a fraction of both CD4^+^ and CD8^+^ T cells, which have infiltrated the tumor stroma, also express PD‐1. Of note, we detected CD8^+^ PD‐1^+^ cells in the stroma as well as in the actual tumor nodules, while CD4^+^ PD‐1^+^ exclusively localized to the tumor microenvironment (Fig. S2C).

Intriguingly, expression of the PD‐1 ligand PD‐L1 in mouse tumors was mainly found in the Sca‐1^+^ and/or CD49f^+^ epithelial compartment of BCC‐like lesions, suggesting cell‐autonomous induction of this immune inhibitory molecule in response to oncogenic Hh/Gli activation in epidermal cells (Fig. 2D), consistent with a recent study showing direct GLI‐mediated PD‐L1 activation in a murine organoid model of Gli2‐driven gastric cancer [59].

Having shown that PD‐1 is upregulated on skin T cells of *Ptch^Δep^* mice, we set out to test whether re‐activation of the antitumor immune response *via* anti‐PD‐1 immune checkpoint inhibition results in tumor regression. For this purpose, we injected TAM‐treated *K14CreER^T^;Ptch^f/f^* with anti‐PD‐1 blocking antibodies (Fig. 2E). However, and unlike in human case studies, anti‐PD‐1 treatment was unable to reduce the tumor burden of *Ptch^Δep^* mice (Fig. 2F and Fig. S3A). Analysis of the immune infiltrate of the skin *via* flow cytometry did not reveal significant alterations in the immune cells following PD‐1 therapy (Fig. S3B). Since the response to immune checkpoint inhibition correlates with mutational burden [45], we hypothesized that the lack of sufficient immunogenic mutations in our murine model of BCC may account for the failure of PD‐1 therapy. Therefore, we performed whole‐exome sequencing (complete mouse exome coverage 49.6 Mb) of mouse BCC‐like lesions (*n* = 3) and control skin (*n* = 3) to survey the mutational landscape of *Ptch^Δep^* tumor lesions. As shown in Table S5, we were unable to identify any mutations in two of three mice. One mouse tumor showed two mutations with unknown immunological consequence (Table S5). Furthermore, analysis of the copy number variations revealed minor negligible chromosomal alterations in all three mice analyzed (Fig. S4). Based on these data, we propose that *Ptch^Δep^* tumors do not express neoantigens and, thus, are likely nonimmunogenic, although we cannot exclude the possibility of epigenetic re‐activation of endogenous retroviral sequences [60]. In summary, the lack of additional mutations underlines the need for next‐generation immunogenic mouse models of HH/GLI‐driven skin cancer to evaluate immunotherapeutic strategies.

To show the relevance of these murine data to human pathophysiology, we performed immunohistochemical staining of human BCC specimen for CD8 and PD‐L1. We subdivided the samples according to their PD‐L1 expression in PD‐L1 low and high (Fig. 2G). Thereby, we revealed that PD‐L1 low samples showed a definable inflammation mostly in the peri‐tumoral regions, while PD‐L1 high samples showed marked immune infiltration of the tumor. Here, double staining of PD‐L1 and CD8 showed a pronounced infiltration of PD‐L1 high BCC tumors with CD8^+^ T cells together with focal PD‐L1 expression on tumor cells in close proximity to CD8^+^ T lymphocytes, suggesting inhibition of cytotoxic T cells by PD‐L1 expressing BCC cells *via* immune checkpoint signaling (Fig. 2G,H). In addition, human BCC expressing high levels of PD‐L1 present a marked increase in infiltrating PD‐1^+^ cells in the tumor microenvironment (Fig. 2H).

### 3.3 Mouse BCC‐like skin displays an altered cytokine and chemokine expression profile

Having shown that largely unmutated *Ptch^Δep^* lesions comprise altered T cell populations in comparison to *Ptch^f/f^* skin, we hypothesized that also the cytokine and chemokine expression profiles differ between tumor lesions and control mice. We, therefore, performed by qPCR analysis and Luminex profiling a systematic mRNA and protein expression analysis of tumor and control skin for known immune‐modulating cytokines and chemokines (for summary, see Table 1). Of note, we found increased levels of several immunosuppressive cytokines such as *Tslp*, transforming growth factor β (*Tgf*β), *Il10,* and *Inos* as well as of pro‐inflammatory cytokines such as *Tnf*α, *Il1*β, *Ifn*γ, and *Il17* in tumor lesions compared to control skin. Furthermore, BCC‐like lesions expressed elevated levels of immune cell attracting chemokines such as *Ccl2*, *Ccl3,* and *Ena78* [61, 62]. We observed similar changes in cytokine/chemokine expression profiles in *SmoM2* BCC mice (Fig. S5). We conclude that persistent Hh/Gli signaling in epidermal cells induces pronounced changes in the expression of immune‐modulatory and chemoattractant factors that contribute to the formation of an immunosuppressive tumor microenvironment, including shifts of T lymphocyte and possibly also myeloid cell populations.

**Table 1:** Cytokine and chemokine expression profiles in murine BCC. nd, not determined.

Gene name	mRNA		Protein	
	Fold change	*P*‐value	Fold change	*P*‐value
Cytokines				
Il10	4.2	0.0523	nd	nd
Tgfβ	4.5	0.0039	nd	nd
Il1β	13.4	0.0024	3.4	0.1036
Ifnγ	7.8	0.0011	nd	nd
Il17	27.5	0.0098	nd	nd
Gmcsf	3.9	0.0104	1	0.9949
Inos	5.6	0.0425	nd	nd
Cox2	2.2	0.0747	nd	nd
Tslp	47.3	0.0710	2.6	0.0010
Tnf	2.2	0.0688	2.6	0.0083
Chemokines				
Mip‐2	nd	nd	1.2	0.6193
Ena78	13.1	0.0003	1.4	0.4529
Gro‐α	nd	nd	1.5	0.3141
Ccl2/Mcp‐1	17	0.0418	4.6	0.0284
Ccl3/Mip‐1α	3.8	0.0125	4.1	0.0161
Ccl7/Mcp‐3	nd	nd	2.5	0.2955
Cxcl10/Ip‐10	nd	nd	1.1	0.9148

### 3.4 Oncogenic HH signaling leads to altered innate immunity in mouse BCC‐like skin

**Fig. 3:**
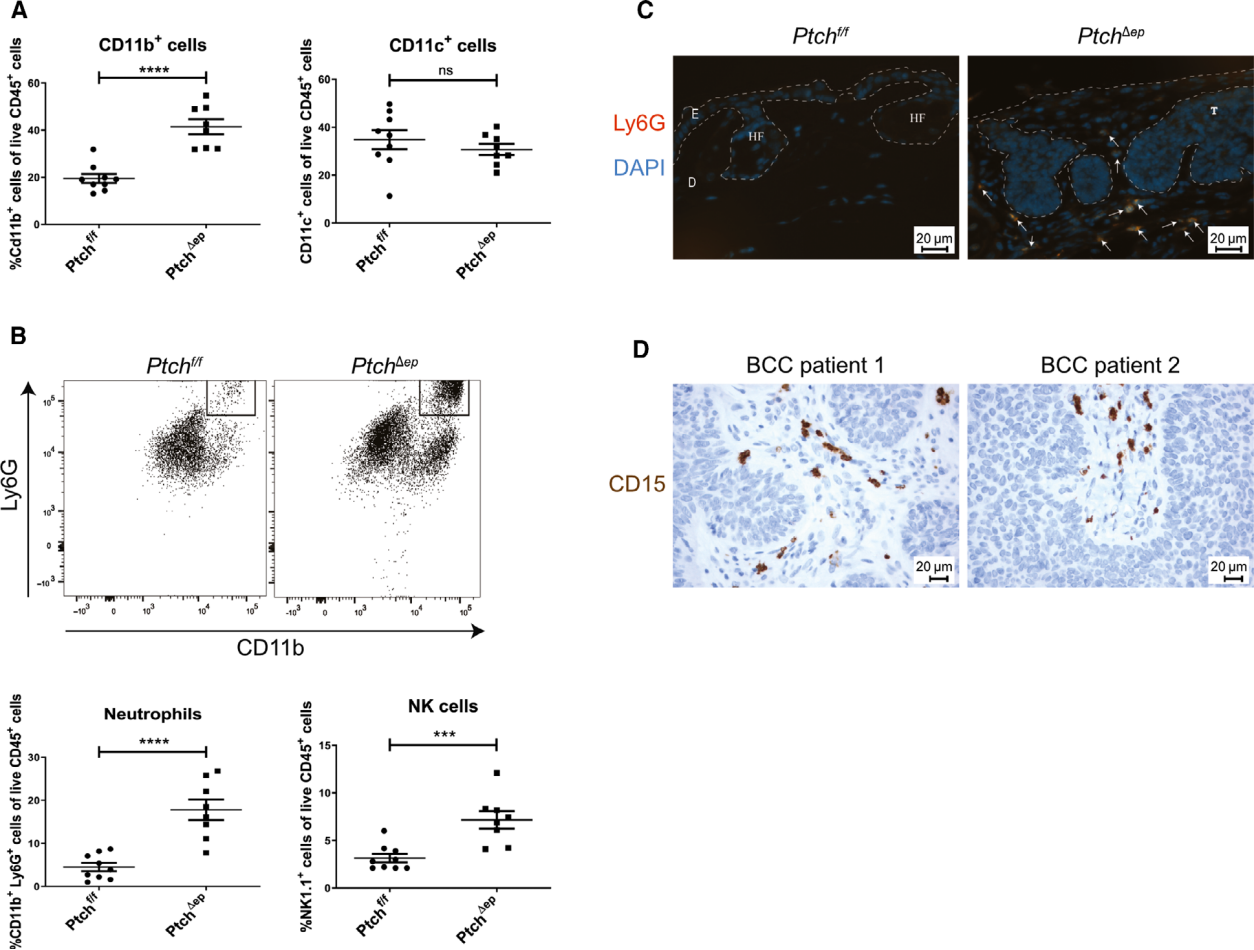
Oncogenic HH signaling leads to altered innate immunity in BCC. (A) Flow cytometry analysis of murine skin for CD11b and CD11c expression in *Ptch^Δep^* (*n* = 8) and *Ptch^f/f^* control (*n* = 9) mice. (B) Representative flow cytometry plots for expression of Ly6G and CD11b (upper panel) and flow cytometry analysis for Ly6G and NK1.1 expression (*Ptch^f/f^* *n* = 9 and Ptch*^Δep^* *n* = 8; lower panel). (C) Immunofluorescence staining of murine ear skin for Ly6G (red) and DAPI (blue). The arrows indicate Ly6G^+^ cells. E: epidermis, D: dermis, T: tumor mass, HF: hair follicle (scale bar 20 µm). (D) Representative immunohistochemical stainings of two human BCC skin sections for the neutrophil marker CD15 (brown) with nuclei stained in blue (scale bar 20 µm). Statistical analysis for all experiments was performed with Student's *t* test. ***P < 0.001 and ****P < 0.0001.

Having demonstrated that Hh/Gli signaling in mouse BCC‐like tumors results in a significant increase in chemokine levels such as Ccl2 and Ccl3—two well‐known chemoattractants for innate immune cells [62, 63]—we next analyzed quantitatively and qualitatively by flow cytometry analysis, whether epidermal Hh pathway activation results in changes of the innate immune cell population. To this end, we gated myeloid cells in skin samples of *Ptch^f/f^* and *Ptch^Δep^* mice by their CD11b and CD11c expression. Intriguingly, we found a pronounced increase in CD11b^+^ cells in mouse skin tumors (Fig. 3A), which we confirmed also in *SmoM2* mice (Fig. S6A). However, *SmoM2* mice showed a strong decrease in CD11c expression (Fig. S6A), while we did not see this prominent effect in *Ptch^Δep^* mice (Fig. 3A).

Further analysis of CD11b^+^ cells using the neutrophil marker Ly6G and the natural killer (NK) cell marker NK1.1 revealed a noticeable recruitment of both innate immune cell populations in *Ptch^Δep^* skin lesions (Fig. 3B). Ly6G^+^ cells were also increased in skin BCC‐like lesions of *SmoM2* mice (Fig. S6B). To better understand the putative role of neutrophils in BCC‐like lesions, we first performed immunofluorescence staining of Ly6G^+^ cells in skin samples of *Ptch^f/f^* and *Ptch^Δep^* mice. We found most of the Ly6G^+^ cells to be located in the peri‐tumoral region with only rare cells within the tumor nest itself (Fig. 3C).

Similarly, staining of human BCC samples for the neutrophil marker CD15 revealed CD15^+^ cells in the stromal compartment of human BCC tumors (Fig. 3D).

**Fig. 4:**
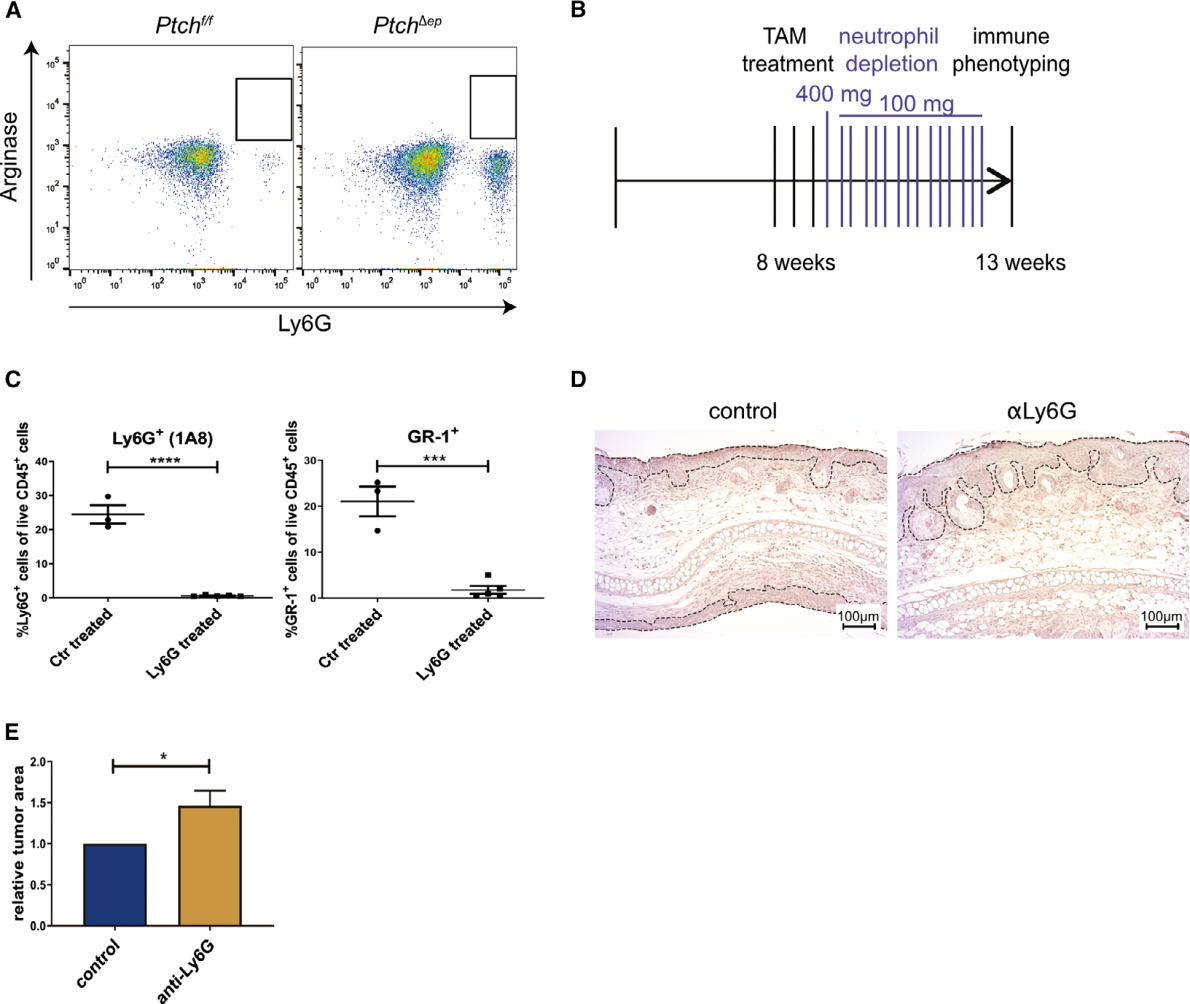
Neutrophil depletion in BCC mice accelerates tumor growth. (A) Representative flow cytometry analysis plots of arginase staining in *Ptch^Δep^* and *Ptch^f/f^* mouse skin (*Ptch^f/f^* *n* = 2 and Ptch*^Δep^* *n* = 4). (B) Treatment schedule for neutrophil depletion in *K14CreER^T^;Ptch^f/f^* mice previously injected with TAM. (C) Flow cytometry analysis of murine skin for Ly6G or Gr‐1 expression verifying efficient depletion of neutrophils (control *n* = 3, anti‐Ly6G *n* = 5). (D) Representative H&E staining comparing tumor development in ear skin of *Ptch^Δep^* mice not depleted (left) or depleted (right) of neutrophils during BCC progression. The dashed lines mark the tumor area (scale bar 100 µm). (E) Quantification of the tumor area in the ear skin of *Ptch^Δep^* mice not depleted (control, *n* = 3) or depleted (anti‐Ly6G, *n* = 3) of neutrophils during BCC progression. Statistical analysis for all experiments was performed with Student's *t* test, and for (E), the error bar indicates the SD. *P < 0.05, ***P < 0.001 and ****P < 0.0001.

Since Fan *et al*. [64] have provided evidence that in *SmoM2* BCC mice Ly6G^+^ CD11b^+^ cells represent myeloid‐derived suppressor cells (MDSCs) with immunosuppressive function, we analyzed Ly6G^+^/CD11b^+^ cells of *Ptch^Δep^* mice for the MDSC marker arginase (for antibody validation see Fig. S7). As shown in Fig. 4A, Ly6G^+^/CD11b^+^ cells of *Ptch^Δep^* mice did not express the MDSC marker arginase, suggesting that tumor lesions of *Ptch^Δep^* mice are infiltrated by neutrophils rather than MDSCs (Fig. 4A). To shed light on the role of tumor‐infiltrating neutrophils in BCC, we next depleted *Ptch^Δep^* mice of neutrophils by injecting anti‐Ly6G depletion antibody (clone 1A8; Fig. 4B) and proved successful depletion of neutrophils in the skin by flow cytometry (Fig. 4C). Intriguingly, depletion of neutrophils enhanced the growth of tumor lesions in *Ptch^Δep^* mice (Fig. 4D,E), suggesting that neutrophils, known for their tumor‐promoting function in a variety of malignant entities [65], may constitute a yet unknown component of the antitumoral immune response in BCC. Future studies are required to better understand the possible anticancer activity of neutrophils in the context of HH/GLI‐driven BCC.

## 4 Discussion

The continuous growth and spread of malignant cells depend on the generation of an immunosuppressive microenvironment to prevent an efficient antitumoral immune response. The restoration of the immune response for instance by the use of checkpoint inhibitors has marked a breakthrough in cancer therapy with yet unprecedented success in responding patients [26]. A high mutational burden of cancer cells has been shown to correlate with the clinical response and success of immunotherapy using checkpoint inhibitors [45]. Given the exceptionally high mutational burden of human BCC [47], we here hypothesized that (a) HH/GLI‐induced human BCC is immunogenic, (b) HH/GLI signaling establishes an immunosuppressive tumor microenvironment, and (c) HH/GLI‐driven BCC is susceptible to immunotherapy. To investigate in detail the poorly defined role of HH/GLI in the process of immune evasion, we used genetic mouse models of BCC that closely mimic the genetics of driver mutations in human BCC. In the present study, we show that active epidermal HH/GLI signaling is a potent inducer of immunosuppressive mechanisms such as the accumulation of Tregs, the upregulation of immune checkpoints and the production of immunosuppressive growth factors and cytokines including IL10 and TGFβ within the tumor lesions. Although we did not determine the *in vivo* source of these immunosuppressive signals, it is well possible that these key immunosuppressive factors derive directly from cancer cells in response to active HH/GLI. This is supported by previous studies showing that Sonic HH/GLI signaling can induce IL10 expression in a murine model of colitis and pancreatitis. Similarly, overexpression of active GLI2 in T cells can cell‐autonomously enhance IL10 and TGFβ expression [66, 67, 68, 69]. In line with the expression of IL10 and TGFβ, we show increased numbers of Treg cells in *Ptch^Δep^* tumors, similar to human BCC, where Omland *et al*. [70] provided evidence that active HH/GLI signaling can induce Treg accumulation along with a strong increase of TGFβ expression. Furthermore, Hanna *et al*. [71] have recently shown that HH/GLI signaling can drive Treg recruitment in breast cancer, while pathway inhibition reduced the number of Tregs in the tumor. Similar results were shown by Otsuka *et al*. for human BCC [72]. The high prevalence of Tregs within the tumor microenvironment is, thus, likely to constitute a major mechanism of immunosuppression by HH/GLI signaling in cancer.

In addition to an increased level of Treg cells, we demonstrated enhanced expression of the immune checkpoint molecules PD‐1 and its cognate ligands PD‐L1 and PD‐L2 in *Ptch^Δep^* mouse tumors, with PD‐L1 expressed preferentially on epithelial tumor cells and PD‐1 on adjacent CD8^+^ and CD4^+^ T cells. This suggests that HH/GLI signaling in BCC can induce PD‐L1 expression on tumor cells and, thereby, contribute to the suppression of infiltrating cytotoxic T cells directed against the tumor cells. Together with previous reports on the role of HH/GLI in gastric cancer showing PD‐L1 upregulation by HH/GLI signaling [59, 73], our findings in BCC suggest that HH/GLI‐mediated induction of the PD‐L1/PD‐1 immune checkpoint may represent a more general mechanism of HH‐mediated immune escape in cancer. Intriguingly, the increased expression in *Ptch^Δep^* lesions of additional immune checkpoints such as Tigit and Tim3 (Fig. 2A) [54] points to a broader involvement of oncogenic HH/GLI signaling in the suppression of cytotoxic T cell responses, increasing the therapeutic opportunities for combination treatments with anti‐HH and immune checkpoint inhibitors.

In agreement with our findings on immune checkpoint expression profiles of murine BCC‐like lesions, first proof‐of‐concept studies support a therapeutic benefit of anti‐PD‐1 treatment for patients with advanced or metastatic and SMO inhibitor‐resistant BCC [27, 28, 74]. These positive initial results underline the importance of predictive mouse models of BCC that allow the evaluation of innovative combination therapies including immunotherapeutics. Thus, our characterization of the immunological alterations in response to Hh/Gli activation in *Ptch^Δep^* and *SmoM2* mouse models is crucial to understand the immune infiltration and cytokine/chemokine expression profiles in BCC‐like skin lesions. It proves the usefulness of these mouse models to study in detail the pathologically relevant changes in the immune microenvironment of HH/GLI‐induced BCC. However, the failure of *Ptch^Δep^* mice to respond to anti‐PD‐1 immune checkpoint inhibitor therapy, which may be due to the absence of immunogenic mutations as shown by whole‐exome sequencing, reveals the limitations of this common BCC mouse model for immunotherapy approaches. Unlike human BCC, murine BCC‐like lesions displayed a moderately reduced number of T cells. We speculate that the decrease of T cells, particularly of CD8^+^ cells, may be a consequence of a lack of neoantigens and of increased levels of immunosuppressive cytokines in the tumor microenvironment, which may induce T cell anergy and apoptosis. The development of immunogenic murine BCC models is, therefore, paramount for future preclinical studies.

By staining human BCC, we revealed that these tumors could be subdivided into tumors with low and high PD‐L1 expression, respectively. In PD‐L1 low tumors, the CD8^+^ T cell infiltration was mostly restricted to the peri‐tumoral regions, while in PD‐L1 high tumors CD8^+^ T cells were located in close proximity to the PD‐L1‐expressing tumor cells. Intriguingly, we detected PD‐1 expression on both CD8 and CD4 T cells in the tumor stroma, including infiltration of the epithelial tumor compartment by CD8^+^PD1^+^ cells. It will be of interest to evaluate whether the stratification of patients into PD‐L1 high/low or into high/low infiltration with CD8^+^PD1^+^/ CD4^+^PD1^+^ T cells results in differential response rates to immune checkpoint blockers. The current lack of patient stratification and predictive markers may explain why the combined treatment of BCC patients with vismodegib and pembrolizumab did not yield a significant therapeutic benefit (trial ID: NCT02690948).

In addition to changes of the T cell populations, we also identified pronounced infiltration of BCC‐like lesions by Ly6G^+^/Arginase^neg^ neutrophils, which was in agreement with the elevated expression of chemokines such as Ccl2 and Ccl3, two potent chemoattractants for myeloid cells [62, 63]. Since the selective depletion of neutrophils accelerated tumor development in *Ptch^Δep^* mice, we conclude that neutrophils, which we found to be mainly located in the tumor periphery, can fulfill antitumorigenic functions in HH/GLI‐driven skin cancer. Although neutrophils are well known for their tumor‐promoting function [35, 75, 76], evidence also suggests a critical contribution of neutrophils to the antitumoral immune response, reflecting their remarkable plasticity [77]. For instance, neutrophils have been shown to enhance MHC‐I expression, thereby increasing the antitumoral response of adaptive immunity. Further, tumor‐associated neutrophils can promote the proliferation of effector T cells and impede tumor progression by the release of pro‐apoptotic TRAIL (reviewed in [65]). Whether these mechanisms account for the tumor‐suppressive activity of neutrophils in murine BCC skin needs to be addressed in future studies. In this context, it is also noteworthy that Fan *et al*. have provided evidence that prenatal epidermal activation of *SmoM2* resulted in BCC‐like lesions enriched for Ly6G^pos^ pro‐tumorigenic MDSCs. Recruitment of MDSCs was inhibited by systemic chemical inhibition of Ccl2 receptor, resulting in reduced tumor growth [64]. However, in contrast to our data and studies on human BCC, *SmoM2* mice with prenatal Hh/Gli activation and MDSC enrichment did not show enhanced Treg numbers, which may point to subtle differences in the immune modulation of the distinct mouse models used.

## 5 Conclusion

Taken together, we conclude that the mere genetic activation of HH/GLI signaling in epidermal cells induces profound changes within the immune microenvironment of BCC‐like lesions, thereby establishing a potent immunosuppressive milieu, which is likely to inhibit the antitumoral immune response against human BCC cells with high mutational burden. Therapeutic strategies directed against immunosuppressive mechanisms activated by HH/GLI including several checkpoint inhibitors warrant further evaluation. Our results also call for next‐generation, immunogenic models of BCC to explore the full therapeutic potential of treatments including HH pathway inhibitors in combination with selected immunotherapeutic drugs.

## Conflict of interest

The authors declare no conflict of interest.

## Author contributions

SG‐G, DO, ABS‐N, DN, IKG, PS, and FA were responsible for the concept and experimental design. SG‐G, DO, and DN performed the experiments. SG‐G, DO, ABS‐N, DN, NZ, IKG, PS, and FA analyzed and interpreted the data. ABS‐N, RW, DN, MW, AR, PH, RG, NZ, MS, IKG, and PS helped to develop the methodology. SG‐G and FA wrote the manuscript. All authors read and approved the final manuscript.

## Ethical approval

Human BCC specimen used for immunohistochemical analysis was analyzed and processed in accordance with the guidelines of the Austrian ethics committee. Animal studies were approved by the institution authorities and by the Federal Ministry of Science, Research and Economy (BMWFW‐66.012/0016‐WF/V/3b/2015 and BMWFW‐66.011/0030‐II/3b/2014).

## Supporting information


**Fig. S1.** Altered immune phenotype of *SmoM2* mice. (A–D) Flow cytometry analysis of (A) CD3^+^ (*Smo^WT^* *n* = 7, *SmoM2* *n* = 6) and CD3^high^ (*Smo^WT^* *n* = 7, *SmoM2* *n* = 7) T cells, (B) CD4 and CD8 T cells in *Smo^WT^* (*n* = 5) and *SmoM2* (*n* = 7) mice. (C, D) mRNA expression level of (C) *Foxp3* and (D) *Pd‐l1* in *Smo^WT^* (*n* = 3) and *SmoM2* (*n* = 3) mice. For *Foxp3* expression analysis mice were harvested ~ 4 weeks (early, *n* = 3) and 12 weeks (late, *n* = 3) post Tamoxifen administration.
**Fig. S2.** BCC tumors reveal co‐expression of PD‐1 with CD4 and CD8 but not FoxP3. (A) Two representative immunohistochemical stainings of human BCC skin sections stained for FoxP3 (red) and PD‐1 (brown), nuclei are stained in blue (scale bar 20 µm). (B) Flow cytometry analysis of PD‐1 expression on CD3^+^ T cells in *Ptch^f/f^* (*n* = 2) and *Ptch^Δep^* (*n* = 4) mice. (C) Representative immunohistochemical stainings of two human BCC skin sections for PD‐1 (brown) with CD4 or CD8 (red), nuclei are stained in blue. White arrowheads indicate representative double positive cells (scale bar 20 µm).
**Fig. S3.** Anti‐Pd‐1 blocking does not reduce skin cancer phenotype of *Patched^Δep^* mice. (A) Quantification of the tumor area in the ear skin of *Ptch^Δep^* mice untreated or treated with anti‐PD‐1 blocking antibodies during BCC progression (*n* = 4 per group). (B) Flow cytometry analysis of the skin after Pd‐1 blocking (*n* = 4 per group).
**Fig. S4.** Mouse tumors exhibit no large structural genetic variations. Representative copy number variation (CNV) plot from mouse #2. CNV analysis reveals no deletions or amplifications.
**Fig. S5.** Altered cytokine and chemokine profile of *SmoM2*. Cytokine and chemokine profiles were determined *via* mRNA expression level of *Smo^WT^* (*n* = 4, for *Il10* and *Ccl2* *n* = 3) and *SmoM2* mice (*n* = 3).
**Fig. S6.** Altered innate immunity in *SmoM2* mice. (A–D) Flow cytometry analysis of (A) CD11b (*Smo^WT^* *n* = 6, *SmoM2* *n* = 5) and CD11c (*Smo^WT^* *n* = 4, *SmoM2* *n* = 3) innate immune cells and (B) GR‐1^+^ (*Smo^WT^* *n* = 4, *SmoM2* *n* = 4) and NK1.1^+^ (*Smo^WT^* *n* = 3, *SmoM2* *n* = 3) cells in *Smo^WT^* and *SmoM2* mice.
**Fig. S7.** Validation of the arginase antibody. Representative flow cytometry plots of bone marrow‐derived macrophages unstimulated or stimulated with Il4, a known inducer of arginase expression.

**Table S1.** Antibodies used for flow cytometry.
**Table S2.** Primer sequences used for qPCR of murine samples.
**Table S3.** qPCR primer list for *SmoM2* mice.
**Table S4.** Antibodies used for immunofluorescence and immunohistochemistry.
**Table S5.** Mutational landscape of tumors from *Ptch^Δep^* mice.

## Data Availability

All reagents and material developed during the study will be made available to the scientific community for nonprofit use upon request.
