# Supplementary figures and images for: Epidermal activation of Hedgehog signaling establishes an immunosuppressive microenvironment in basal cell carcinoma by modulating skin immunity
Source: Mol Oncol. 2020 Jul 21;14(9):1930–46. doi: 10.1002/1878-0261.12758 (PMC7463314; doi:10.1002/1878-0261.12758)

a

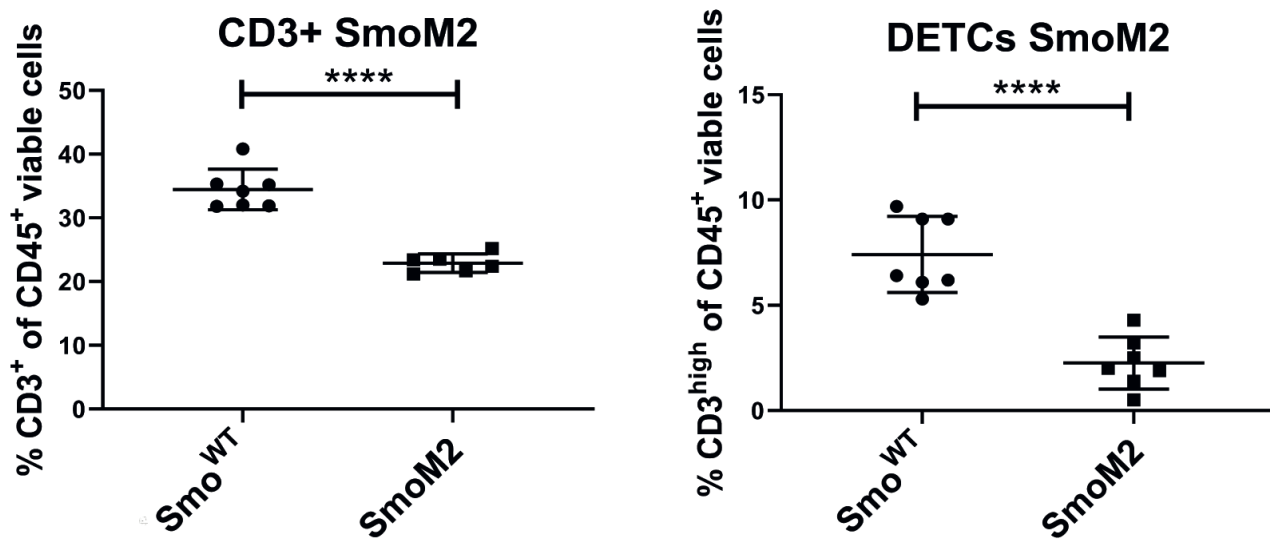

b

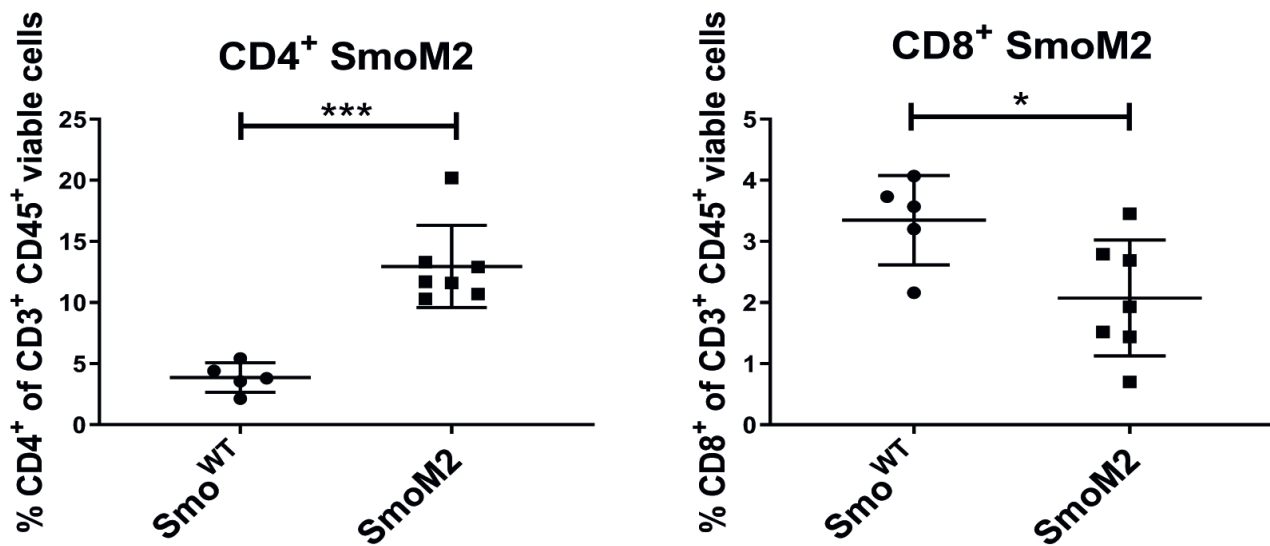

c

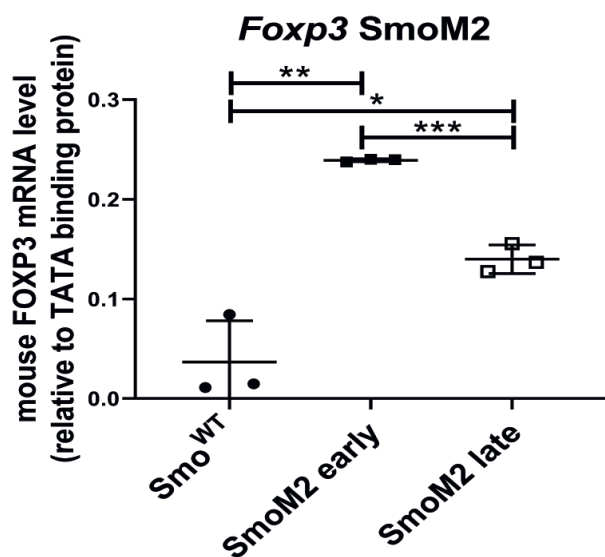

d

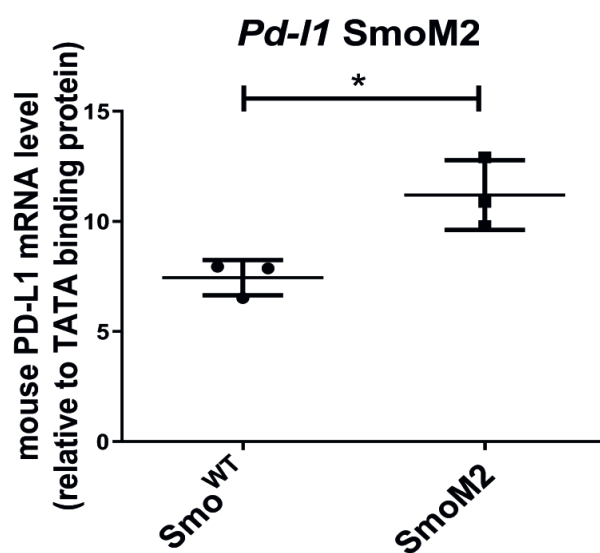

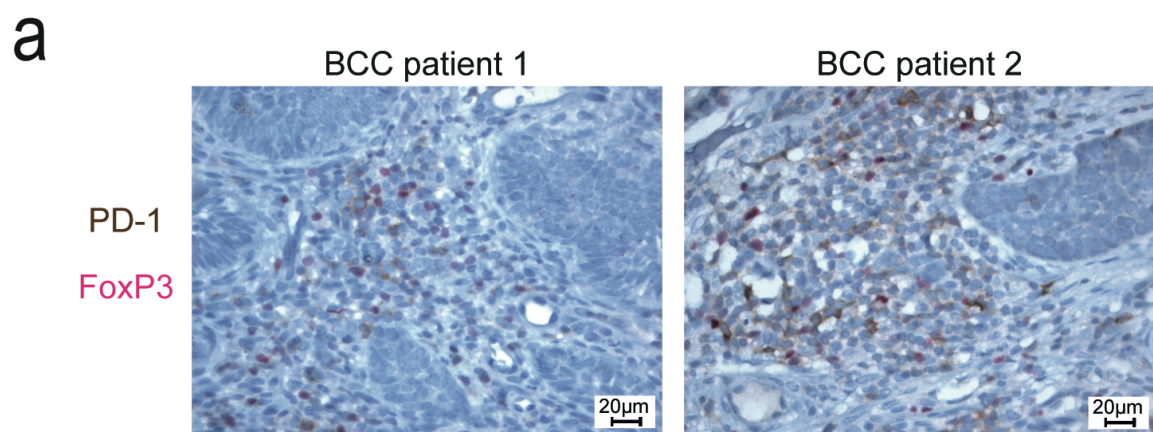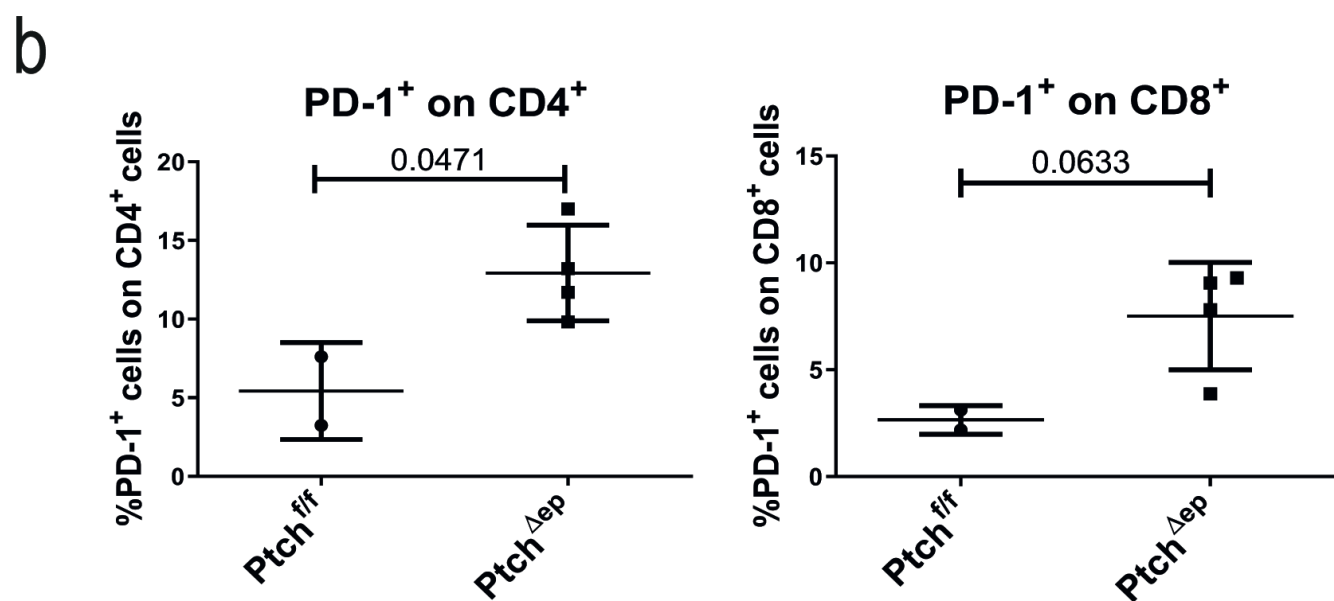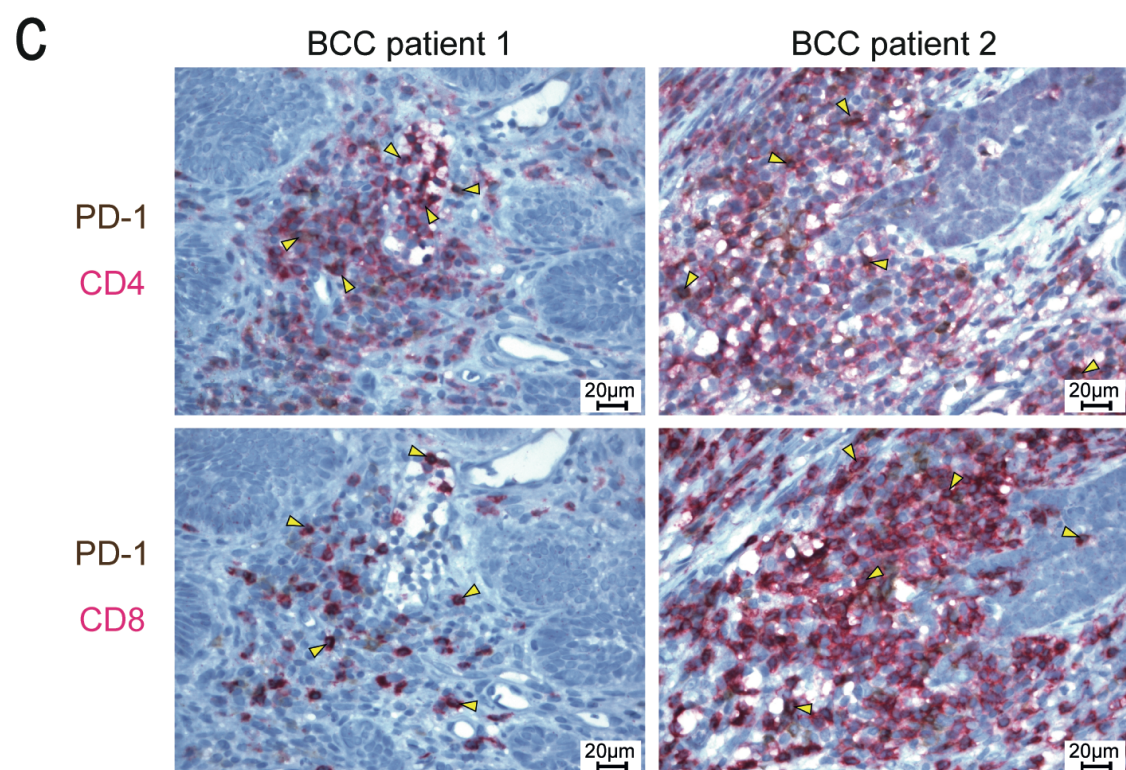

a

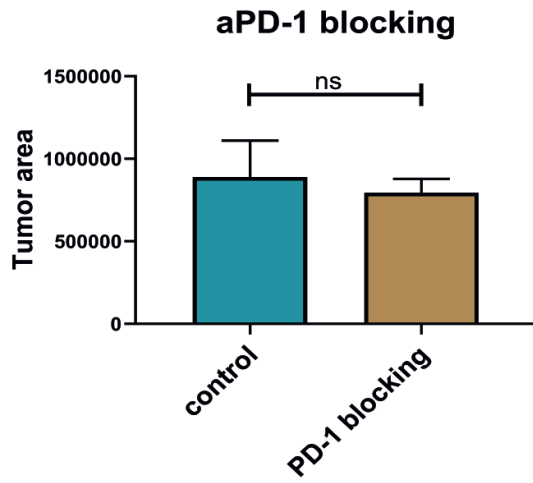

b

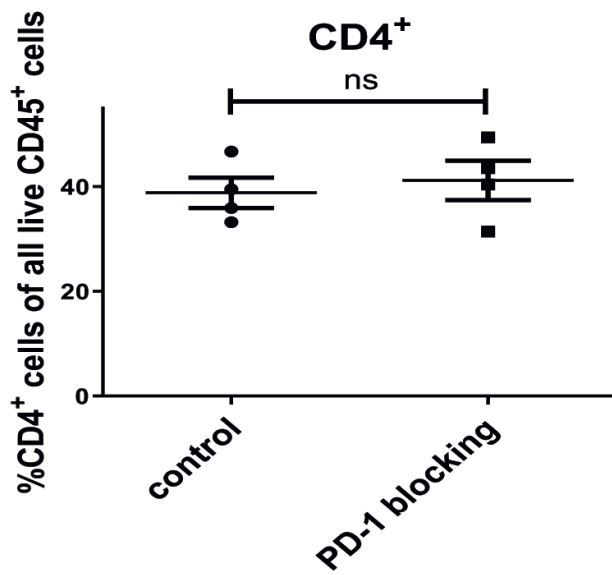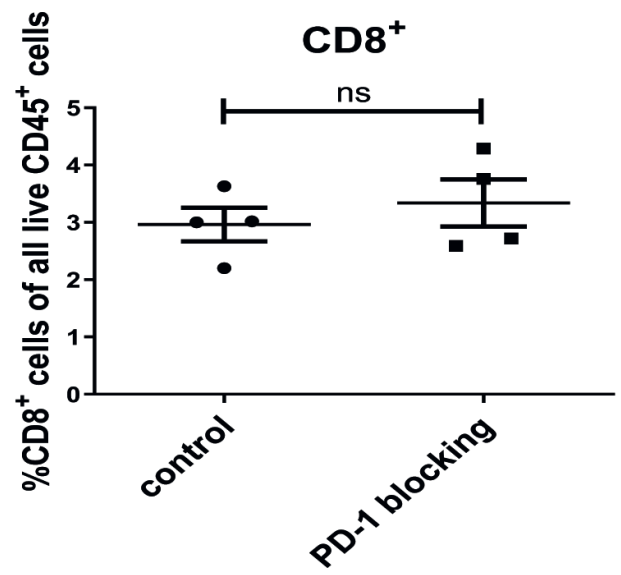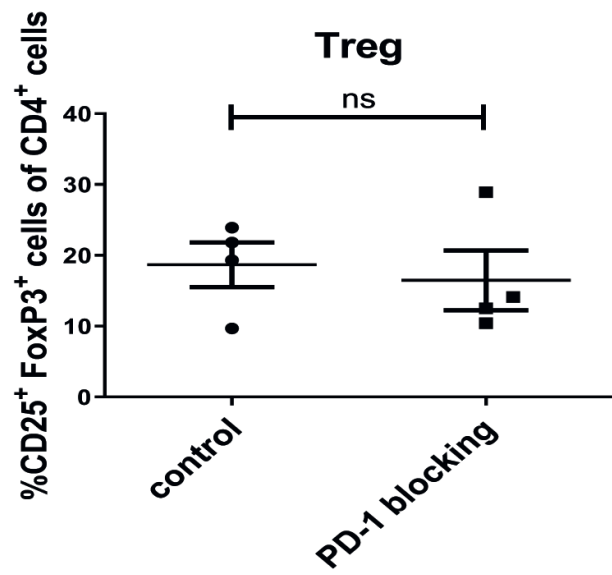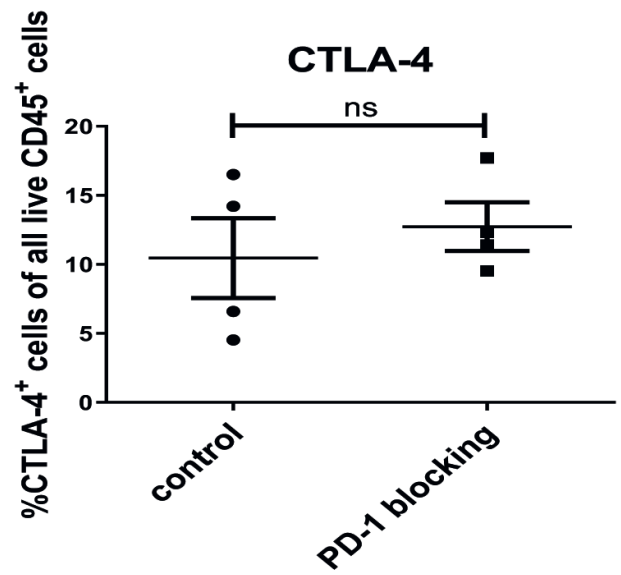

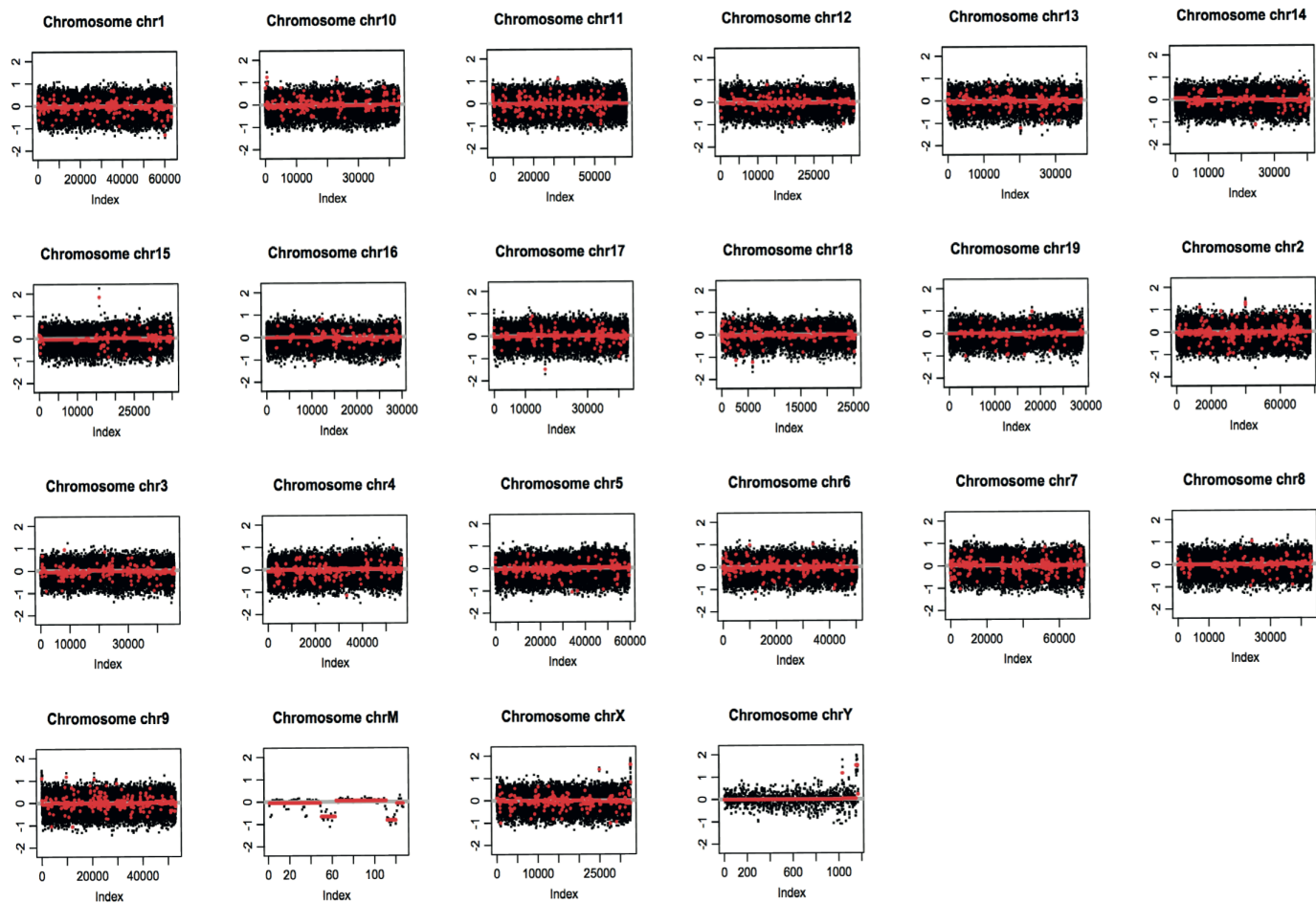

Grund-Groeschke et al. Figure S4

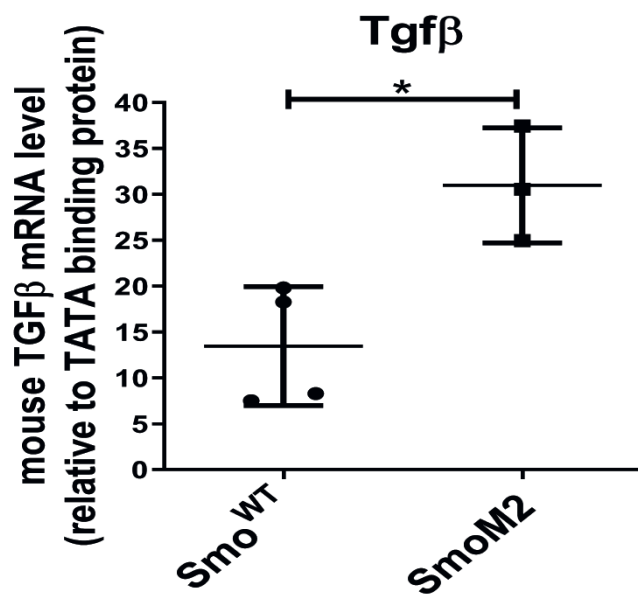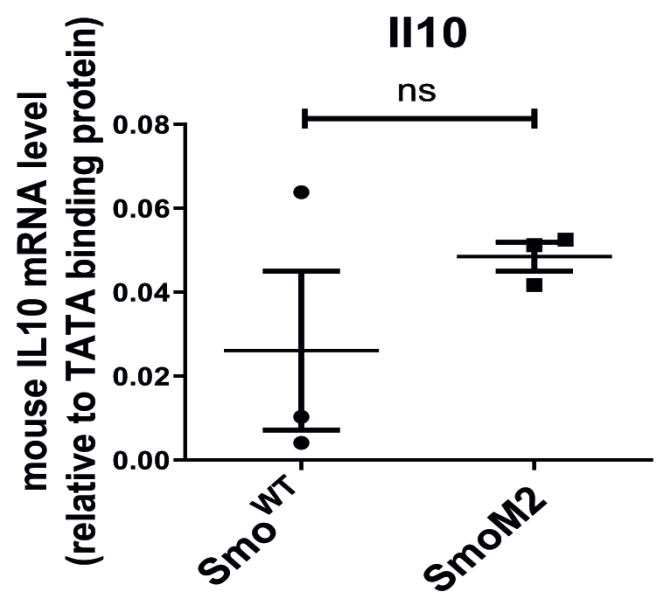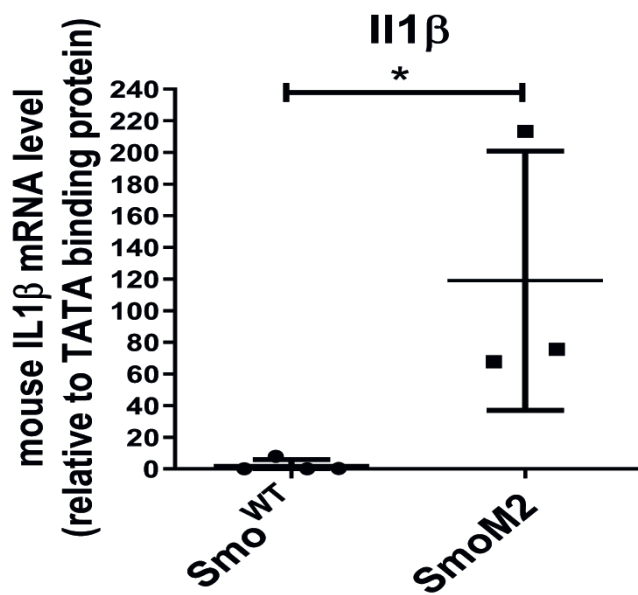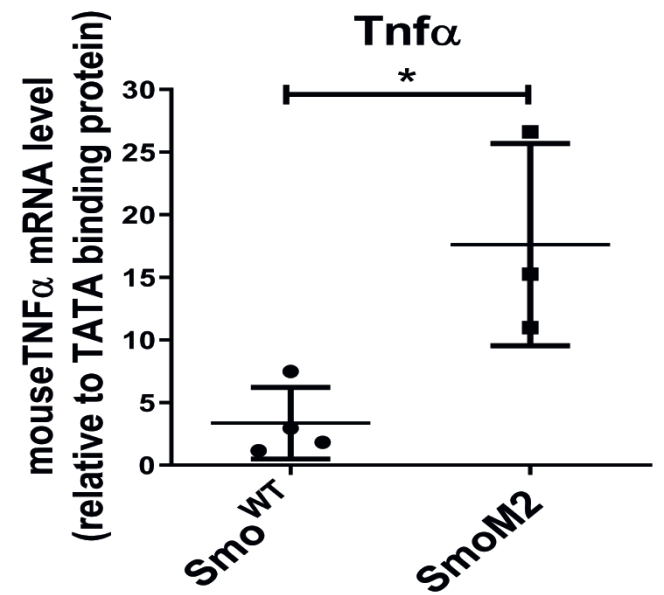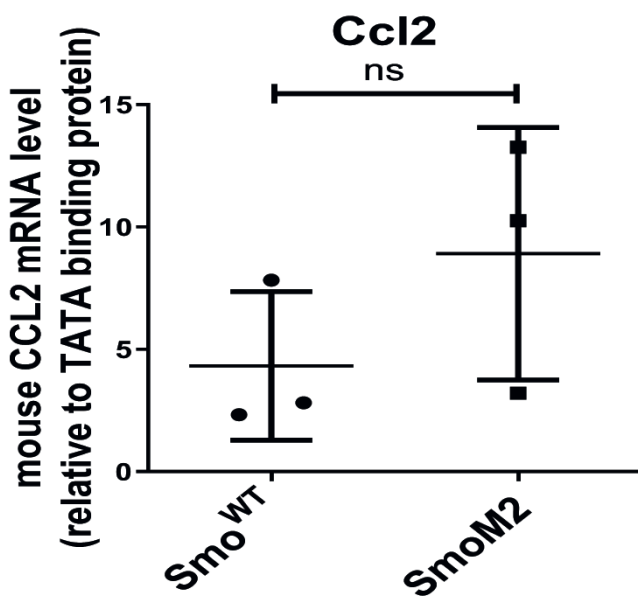

a

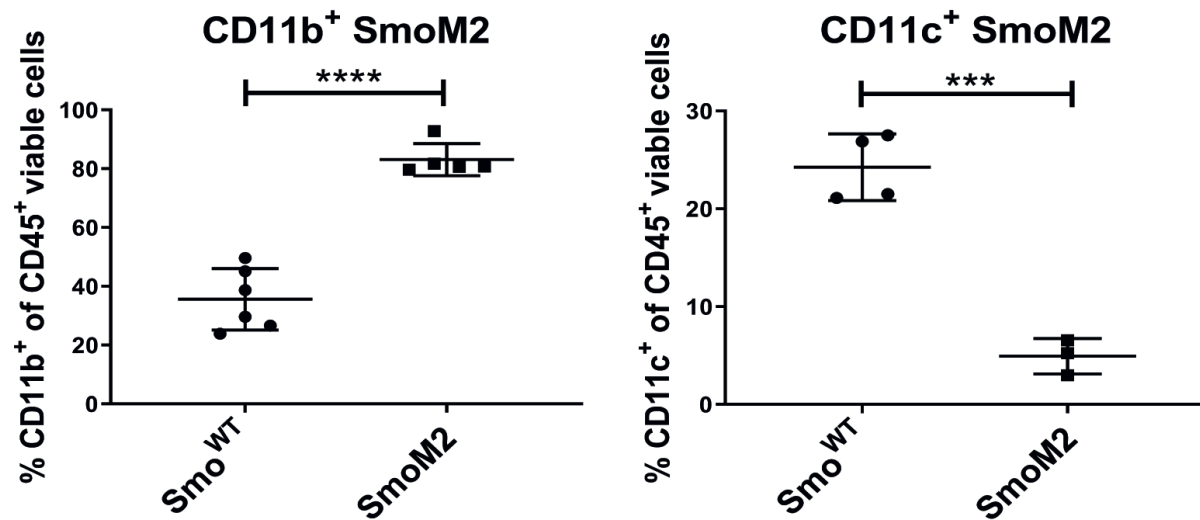

b

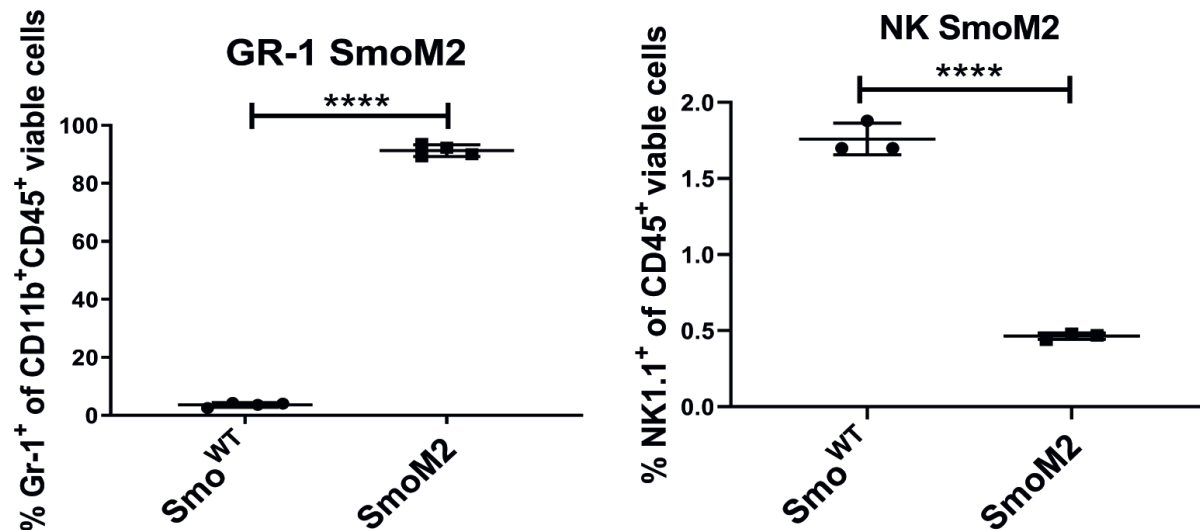

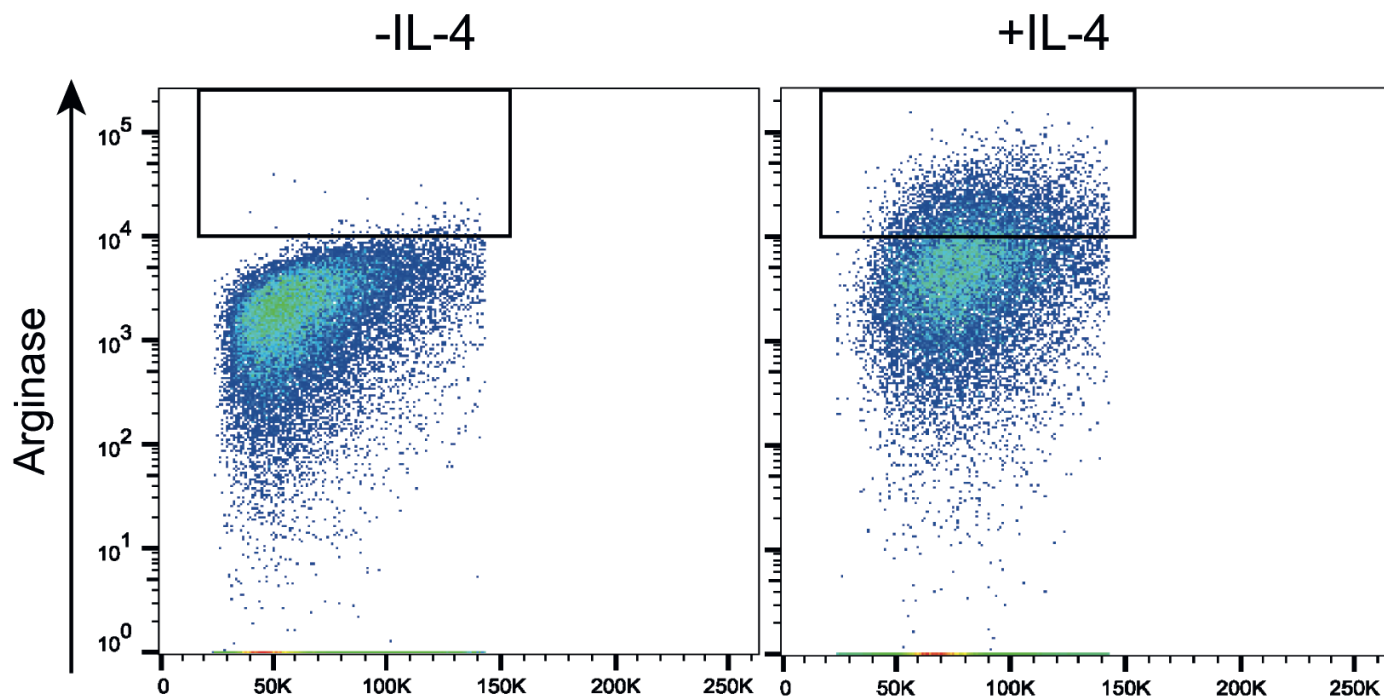

Grund-Gröschke et al. Figure S7

Supplement: Supplementary file 1 — Fig. S1. Altered immune phenotype of SmoM2 mice. (A–D) Flow cytometry analysis of (A) CD3+ (SmoWT n = 7, SmoM2 n = 6) and CD3high (SmoWT n = 7, SmoM2 n = 7) T cells, (B) CD4 and CD8 T cells in SmoWT (n = 5) and SmoM2 (n = 7) mice. (C, D) mRNA expression level of (C) Foxp3 and (D) Pd‐l1 in SmoWT (n = 3) and SmoM2 (n = 3) mice. For Foxp3 expression analysis mice were harvested ~ 4 weeks (early, n = 3) and 12 weeks (late, n = 3) post Tamoxifen administration. Fig. S2. BCC tumors reveal co‐expression of PD‐1 with CD4 and CD8 but not FoxP3. (A) Two representative immunohistochemical stainings of human BCC skin sections stained for FoxP3 (red) and PD‐1 (brown), nuclei are stained in blue (scale bar 20 µm). (B) Flow cytometry analysis of PD‐1 expression on CD3+ T cells in Ptchf/f (n = 2) and PtchΔep (n = 4) mice. (C) Representative immunohistochemical stainings of two human BCC skin sections for PD‐1 (brown) with CD4 or CD8 (red), nuclei are stained in blue. White arrowheads indicate representative double positive cells (scale bar 20 µm). Fig. S3. Anti‐Pd‐1 blocking does not reduce skin cancer phenotype of PatchedΔep mice. (A) Quantification of the tumor area in the ear skin of PtchΔep mice untreated or treated with anti‐PD‐1 blocking antibodies during BCC progression (n = 4 per group). (B) Flow cytometry analysis of the skin after Pd‐1 blocking (n = 4 per group). Fig. S4. Mouse tumors exhibit no large structural genetic variations. Representative copy number variation (CNV) plot from mouse #2. CNV analysis reveals no deletions or amplifications. Fig. S5. Altered cytokine and chemokine profile of SmoM2. Cytokine and chemokine profiles were determined via mRNA expression level of SmoWT (n = 4, for Il10 and Ccl2 n = 3) and SmoM2 mice (n = 3). Fig. S6. Altered innate immunity in SmoM2 mice. (A–D) Flow cytometry analysis of (A) CD11b (SmoWT n = 6, SmoM2 n = 5) and CD11c (SmoWT n = 4, SmoM2 n = 3) innate immune cells and (B) GR‐1+ (SmoWT n = 4, SmoM2 n = 4) and NK1.1+ (SmoWT n [file MOL2-14-1930-s001.pdf]
