# Supplementary material for: Epidermal activation of Hedgehog signaling establishes an immunosuppressive microenvironment in basal cell carcinoma by modulating skin immunity
Source: Mol Oncol. 2020 Jul 21;14(9):1930–46. doi: 10.1002/1878-0261.12758 (PMC7463314; doi:10.1002/1878-0261.12758)
Supplement: Supplementary file 2 — Table S1. Antibodies used for flow cytometry. Table S2. Primer sequences used for qPCR of murine samples. Table S3. qPCR primer list for SmoM2 mice. Table S4. Antibodies used for immunofluorescence and immunohistochemistry. Table S5. Mutational landscape of tumors from PtchΔep mice. [file MOL2-14-1930-s002.docx]

**Supplementary Tables**

| **Suppl. Table S1: Antibodies used for flow cytometry** | | | | |
| --- | --- | --- | --- | --- |
| **Antibody** | **Clone** | **Company** | **Dilution** | **Identifer** |
| CD45 | 30-F11 | Thermo Scientific | 1:1500 | 47-0451 |
| CD11b | M1/70 | Thermo Scientific | 1:500 | 69-0112 |
| Ly-6G | 1A8 | Thermo Scientific | 1:500 | 17-9668 |
| CD11c | N418 | Thermo Scientific | 1:200 | 25-0114 |
| NK-1.1 | PK136 | Thermo Scientific | 1:200 | 12-5941 |
| CD3 | 145‑2C11 | Thermo Scientific | 1:300 | 45-0031 |
| CD4 | GK1.5 | Thermo Scientific | 1:200 | 11-0041 |
| CD4 | GK1.5 | Thermo Scientific | 1:2000 | 12-0041 |
| CD4 | GK1.5 | Thermo Scientific | 1:300 | 48-0041 |
| CD8a | 53-6.7 | Thermo Scientific | 1:400 | 25-0081 |
| CD8a | 53-6.7 | Tonbo Biosciences | 1:400 | 20-0081 |
| γδTCR | eBioGL3 | Thermo Scientific | 1:600 | 12-5711 |
| FoxP3 | FJK‑16s | Thermo Scientific | 1:300 | 48-5773 |
| CD274/PD-L1 | MIH5 | Thermo Scientific | 1:500 | 12-5982 |
| CD49f | eBioH3 | Thermo Scientific | 1:500 | 25-0495 |
| Ly‑6A/E | D7 | Thermo Scientific | 1:1000 | 62-5981 |
| CD279/PD-1 | J43 | Thermo Scientific | 1:200 | 11-9985 |
| CD25 | PC61.5 | Tonbo Biosciences | 1:500 | 12-0251 |
| Arginase | polyclonal | R&D Systems | 1:20 | IC5868P |
| CD45 | 30-F11 | Biolegend | 1:100 | 103138 |
| CD4 | GK1.5 | Biolegend | 1:100 | 100408 |
| CD8 | 53-6.7 | Biolegend | 1:100 | 100752 |
| CD3 | 17A2 | Biolegend | 1:100 | 100234 |
| CD11c | N418 | Biolegend | 1:100 | 117318 |
| Gr1 | RB6-8C5 | BD | 1:2500 | 562709 |
| CD11b | M1/70 | Biolegend | 1:200 | 101228 |
| NK1.1 | PK136 | Biolegend | 1:100 | 108728 |
| CD45 | 30-F11 | Biolegend | 1:100 | 103138 |

| **Suppl. Table S2: Primer sequences used for qPCR of murine samples** | | |
| --- | --- | --- |
| **Gene name** | **Forward primer (5’-3’)** | **Reverse primer (5’-3’)** |
| Rplp0 | ATAACCCTGAAGTGCTCGACAT | CCATTGATGATGGAGTGTGG |
| Gli1 | CACCGTGGGAGTAAACAGGCCTTCC | CCAGAGCGTTACACACCTGCCCTTC |
| Gli1 | CACATCAACAGTGAGCATATCCA | GTGAATAGGACTTCCGACAGC |
| Il17 | TCTCTGATGCTGTTGCTGCTGCTGA | GTCCAGCTTTCCCTCCGCATTGAC |
| Il10 | CTCCTAGAGCTGCGGACTGCCTTCA | CTTCACCTGCTCCACTGCCTTGCTC |
| Tgf-b | ATGAACCGGCCCTTCCTGCTCCT | CAGAAGTTGGCATGGTAGCCCTTGG |
| Nos2 | CCTGCTTTGTGCGAAGTGTCAGTGG | TCTCTTGCGGACCATCTCCTGCATT |
| Il1b | TACAAGGAGAACCAAGCAACGACAAAATACC | AGGGTGGGTGTGCCGTCTTTCAT |
| Ifng | TGAAAGACAATCAGGCCATCAGCA | CAGCAGCGACTCCTTTTCCGCTTC |
| Ccl2 | CCAGCTCTCTCTTCCTCCACCACCA | ACCCATTCCTTCTTGGGGTCAGCAC |
| Ccl3 | GCAGCAGCGAGTACCAGTCCCTTTTC | TCTTCCGGCTGTAGGAGAAGCAGCA |
| Pd1 | GCAATCAGGGTGGCTTCTA | GTTCCAGTTCAGCATAAGATCCTC |
| Pdl1 | CCGGACAGAGGGGATGCTTCTCA | TGTGGAGGATGTGTTGCAGGCAGTT |
| Pdl2 | GCAAAGTGAAAGAGCCACCCTGCTG | GCTGCACCTCCCCTGTACCTGGA |
| Tim3 | TACAGTTCCCTGGTCTTATGAATGA | GTCTGTGTCTCTGAACCATTTCTCT |
| Tigit | ATGGCTGCTGTGCTGGGACTCATTT | TTCCATTCCTGTGGCTCCGCTTCT |
| Lag3 | ACACCTGTAGCATCCATCTGC | CCACACAAATCTTTCCTTTCCAG |
| Cd226 | TCCTGCTTGTTTCATGCTTTCCCAAAT | TCTACCTGATGGGGCTGGACTTTTTCC |
| Cd96 | ATACCATCATCAGTACAACCACAGA | ACCGATACCATTTTCTTACTCCAAG |

| **Suppl. Table S3: qPCR primer list for *SmoM2* mice** | |
| --- | --- |
| **Gene id** | **Assay number *** |
| Il10 | Mm00439614_m1 |
| Tnf | Mm00443258_m1 |
| Ccl2 | Mm00441242_m1 |
| Tgfb | Mm01178820_m1 |
| Il1b | Mm00434228_m1 |
| Pd-l1 | Mm03048248_m1 |
| Foxp3 | Mm00475162_m1 |

* All primers for analysis of *SmoM2* mice were obtained from Thermo Scientific/Applied Biosystems.

| **Suppl. Table S4: Antibodies used for immunofluorescence and immunohistochemistry** | | | |
| --- | --- | --- | --- |
| **Antibodies** | **Source** | **Identifier** | **Dilution** |
| \| Rabbit anti-PD-1 \| \| --- \| | Cell Signaling | 84651 | 1:100 |
| Rabbit anti-FoxP3 | Cell Signaling | 12653 | 1:100 |
| Rat anti-Ly6G | Biolegend | 127601 | 1:1000 |
| Rabbit anti-CD8 | Cell Signaling | 98941 | 1:200 |
| Goat anti-Rabbit IgG (H+L), Alexa Fluor 488 | Thermo Fisher | A-11008 | 1:1000 |
| Goat anti-Rat IgG (H+L), Alexa Fluor 555 | Thermo Fisher | A-21434 | 1:1000 |
| CD4 (rabbit monoclonal) (clone sp35) | Ventana | 790-4423 | Ready to use |
| CD8 (rabbit monoclonal) (clone sp3) | Ventana | 790-4460 | Ready to use |
| PD-L1 (mouse monoclonal) (clone 22C3) | Agilent DAKO | C3653 | dilution 1:30 |
| PD1 (mouse monoclonal) (clone NAT105) | Cell Marque | CMC48950041 | Ready to use |
| FOXP3 (mouse monoclonal) (clone 236A/E7) | Abcam | ab20034 | dilution 1:100 |
| CD15 (mouse monoclonal) (clone mma) | Ventana | 760-2504 | Ready to use |

| **Suppl. Table S5: Mutational landscape of tumors from *Ptch****^Δ^****^ep^* mice*** | | | | | |
| --- | --- | --- | --- | --- | --- |
| **chromosome** | **Position** | **mutation type** | **Mutation** | **consequence** | **Gene** |
| chr6 | 91504069 | base exchange | C -> G | D274H | Xpc |
| chr18 | 24601227 - 24601244 | deletion | TGCTCGTGGTCCGAGTGA | frame shift | Slc39a6 |

* Table shows mutations found by whole exome sequencing in one out of three *Ptch^Δep^* mice. Chr= chromosome.
